# Quantum circuit complexity and unsupervised machine learning of topological order

**DOI:** 10.1038/s41467-026-71283-5

**Published:** 2026-04-08

**Authors:** Yanming Che, Clemens Gneiting, Xiaoguang Wang, Franco Nori

**Affiliations:** 1 Department of Physics, University of Michigan, Ann Arbor, Michigan USA; 2 Center for Quantum Computing, RIKEN, Wako-shi, Saitama Japan; 3 Zhejiang Key Laboratory of Quantum State Control and Optical Field Manipulation, Department of Physics, Zhejiang Sci-Tech University, Hangzhou, China

**Keywords:** Quantum information, Computational science, Information theory and computation, Topological matter

## Abstract

Enabling the discovery of unknown quantum many-body phases of matter remains a fundamental challenge in machine learning for quantum physics. Here, inspired by the close relationship between Kolmogorov complexity and unsupervised machine learning, we explore quantum circuit complexity as a pivot to build intuitive and efficient unsupervised machine learning for topological order in quantum many-body systems. We argue that Nielsen’s quantum circuit complexity serves as an intrinsic informational distance between topological quantum states that results in interpretable manifold learning. To span a bridge from conceptual power to practical applicability, we present two theorems that connect Nielsen’s quantum circuit complexity of quantum path planning with quantum Fisher complexity (Bures distance) and entanglement generation, respectively. The resulting kernel functions demonstrate superior performance and enhanced interpretability in numerical multiqubit experiments. Our results establish connections between key concepts of quantum computation, quantum complexity, quantum metrology, and machine learning of topological quantum order.

## Abstract (web summary)

Quantum circuit complexity is a central concept in quantum information and field theory. Here, the authors use quantum circuit complexity to construct an approach for unsupervised machine learning of topological order in quantum many-body systems in an interpretable and efficient manner.

## Introduction

The discovery of topological phases of matter has opened a new chapter in modern physics, ranging from symmetry-protected band topologies with short-range quantum entanglement, such as topological insulators and superconductors^1,2^, to topological quantum orders^3–10^ featured by distributed non-local quantum entanglement, such as in Kitaev’s toric code^7,9^. Defying Landau’s paradigmatic approach, where phases and phase transitions are identified through local order parameters (that is, linear functionals of the density matrix of the phase and a local observable), topological phases encode the phase information in global –topological– system properties. This defining difference renders the detection and measurement of topological order challenging for both generic simulation and experimental demonstration.

Recent progress has shown the potential of machine learning for classifying topological phases of matter^11–34^. In particular, the supervised approach to quantum many-body physics problems, which can resort to well established theories of learnability and generalizability^35–37^, has resulted in the formulation of rigorous guarantees^38–47^. However, in many practical scenarios, where a priori knowledge of the different phases of matter (and accordingly their labels) does not exist, only unsupervised machine learning can provide access to the desired information. Despite the practical relevance of unsupervised learning, a rigorous theory, tailored to topological quantum order and offering both interpretability and generalizability, has not yet been fully explored.

One prevalent approach to unsupervised learning is manifold learning^48–50^, where the data is assumed to be distributed on a manifold characterized by an intrinsic distance metric. It is followed by a nonlinear mapping of the data manifold into a low-dimensional Euclidean space while preserving the original similarities and structures. Among many manifold-learning algorithms, diffusion map^51,52^, t-SNE^50^, and kernel principal component analysis (PCA)^53,54^ require a kernel function constructed from the intrinsic distance. The kernel can be used either to build a probabilistic graph representation of the data manifold (e.g., in the diffusion map or the t-SNE), or for PCA in the nonlinear feature space (e.g., in the kernel PCA). Alternatively, the intrinsic distance metric itself can be applied directly to non-kernel manifold learning, such as the Isomap^48^ and the metric-multidimensional scaling (metric-MDS)^55,56^. In the context of topological quantum order, the basic task then is to formulate a distance metric that encodes the nonlinear and non-Euclidean features of topological quantum states and represents topological similarities or distances by exploiting the primitive notion of topological equivalence. Topologically equivalent quantum states, by definition, can be continuously transformed into each other via a smooth unitary transformation generated by a local Hermitian operator with bounded operator norm^4^. In this spirit, a similarity measure based on a path-finding algorithm has been proposed in ref. ^24^ and has shown good performance in clustering symmetry-protected band topologies, which are characterized by short-range quantum entanglement. Other topological similarity measures, that focus on the closing of the spectral gap at topological transitions, have also been proposed and demonstrated^23,57^.

Despite the elegance of these methods in identifying band topologies, the increasing complexity and quantum entanglement in strongly interacting quantum many-body systems may undermine the efficiency and performance of these algorithms. For instance, the path-finding algorithm, when applied to interacting lattice models in order to identify a smooth unitary path generated by a local operator while keeping the parent Hamiltonian gapped, can be principally inconclusive due to the undecidability of the spectral gap for generic quantum many-body Hamiltonians^58^.

**Fig. 1:**
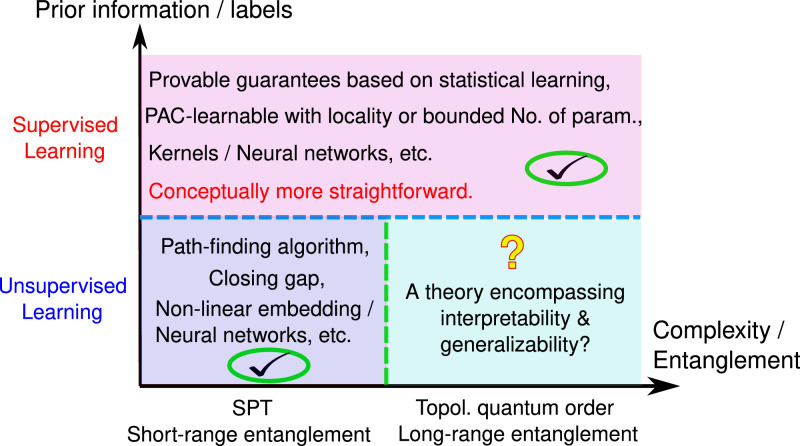
Landscape of machine learning topological phases of matter. Supervised learning, whose efficiency guarantees have been extensively investigated^38–47^, requires prior information or labels. Under the constraint of geometric locality^39,41^ or with a bounded number (No.) of parameters (param.)^40^, provably efficient machine learning of quantum many-body systems has been established, e.g., in the framework of probably approximately correct (PAC)-learnable^35,40^. Supervised learning is conceptually more straightforward due to the existence of well-established statistical learning theories. In contrast, unsupervised learning, whose theory is more complex and less addressed, becomes practically relevant when prior labels are not available. The symmetry-protected topological (SPT) order features short-range entanglement, while the topological quantum order exhibits long-range entanglement. Unsupervised learning with the path-finding algorithm^24^ and kernels focusing on closing gaps at topological transitions^23,57^, respectively, have shown good performance and interpretability for SPT orders or band topologies. However, the direct generalization of these methods to more complex quantum systems with increased complexity and quantum entanglement faces challenges, due to the intrinsic hardness of identifying (or even the undecidability^58^ of) the spectral gap for generic quantum many-body Hamiltonians (See more elaborations and references in the introduction of the main text). Note that reinforcement learning for quantum systems is not included in this landscape.

The inherent problems of previous manifold-learning approaches call for a new similarity ansatz, an ansatz which is able to capture the topological distance and entanglement patterns at different scales while at the same time remaining interpretable and efficiently computable (see Fig. 1 for a schematic of the current state regarding the application of machine learning to the classification of topological phases of matter). To this end, we here adopt the alternative but equivalent perspective that two topologically equivalent multi-qubit states can be transformed into each other via a constant-depth quantum circuit composed of geometrically local quantum gates^3,4^, i.e., the smooth unitary transformation that connects them can be decomposed into a shallow quantum circuit. This implies that it is algorithmically trivial or cheap to generate one quantum state given access to another in the same topological class by fully exploiting the shared structure and entanglement patterns between them.

The correspondence between topological equivalence of quantum states and shallow quantum circuit transformations indicates that, a complexity measure may be related to the topological distance. This is similar to the situation with Kolmogorov complexity, which represents an informational distance between two strings or objects^59^, and may offer a basis for a theoretically optimal solution to unsupervised machine learning (an idea, to the best of our knowledge, first formulated in a talk by Ilya Sutskever). We then assert that the (conditional) generation and prediction of quantum states with the minimal quantum circuit cost may offer a theoretically optimal solution to the unsupervised learning of topological order. The complexity of the generating circuit can be interpreted as a quantum informational distance measure that captures the entanglement change at different scales. Specifically, we propose to use Nielsen’s quantum circuit complexity (QCC) of the unitary path connecting two quantum states as a suitable distance measure that can be used for the unsupervised manifold learning of topological order. While the QCC is already a central concept in quantum information theory^60–65^, quantum field theory^66–69^ and quantum gravity^70–74^, its connection with, and relevance for, the unsupervised machine learning of topological phases of matter has remained mostly unexplored.

In this work, we use the QCC as a pivotal tool to understand and construct unsupervised machine learning of topological order for quantum many-body systems, in an interpretable and efficient manner. Although the exact QCC is generically intractable, we identify various quantities which are upper bounded by the QCC while preserving useful information and remaining implementable, and which thus can be efficiently utilized for classifying topological quantum many-body phases. Specifically, we establish entanglement-based and fidelity-based similarities / kernels, respectively. Numerical experiments with a bond-alternating XXZ qubit chain and Kitaev’s toric code model demonstrate the excellent performance of these methods.

## Results

### Quantum path planning, topological order, and quantum circuit complexity

As mentioned above, unsupervised machine learning of band topology has been extensively investigated. Band topologies, or the more general symmetry-protected topological (SPT) orders, such as those exhibited by topological insulators, are characterized by the absence of long-range quantum entanglement. This implies that, if the constraint of preserving the symmetry is dropped, SPT quantum states can be transformed smoothly into product states. In contrast, topological quantum order refers to quantum states with long-range quantum entanglement^4^; for instance, the ground state of Kitaev’s toric code exhibits topological quantum order that manifests in non-zero topological entanglement entropy^5,6^ and global logical operations^7^, while SPT phases are confined to short-range entanglement. Thus, the task of identifying topological quantum order with unsupervised learning presupposes to construct similarity measures with feature maps that capture quantum entanglement at a variety of scales. For this purpose, we start by defining the quantum path planning between two multi-qubit quantum states, which lays the ground for our search for a distance measure of clustering topological orders.

**Definition 1** [Quantum path planning (QPP).] The QPP problem is defined as: Given two arbitrary pure *n*-qubit quantum states *ρ*_0_ and *ρ*_1_, find continuous paths *ρ*(*s*) = *U*(*s*)*ρ*_0_*U*^†^(*s*) (*s* ∈ [0, 1]) that connect *ρ*_0_ and *ρ*_1_, where the generator *G*(*s*) of *U*(*s*) is a hermitian operator, with the operator norm ∥∂_*s*_*U*(*s*)∥_*∞*_ = ∥*G*(*s*)∥_*∞*_ < + *∞ *(∀ *s* ∈ [0, 1]). In general, we have that $U(s)\in {{{\rm{SU}}}}\left({2}^{n}\right)$ and $iG(s)\in {\mathfrak{su}}\left({2}^{n}\right)$, with the unitary given by

1 $$U(s)={{{\mathcal{P}}}}\,\exp \left(-i{\int }_{0}^{s}G(\tau ){{{\rm{d}}}}\tau \right),$$

where *U*(*s* = 0) = Id is the identity and ${{{\mathcal{P}}}}$ denotes the path ordering operator. The variable *s* can be the normalized time *t* (for instance, *s* = *t*/*T*, where *T* is the total evolution time for the state generation). In practical implementation, the QPP can involve finding the optimal path under various constraints.

Note that, for two topologically equivalent gapped quantum states, the generator *G*(*s*) in the above QPP must be additionally a geometrically local operator, which underlies why the two quantum states can be transformed into each other via a constant-depth quantum circuit^4^ (See the following definition).

**Definition 2** (Topological equivalence^3,4^.) We say that two gapped ground states *ρ*_0_ and *ρ*_1_ of local Hamiltonians *H*_0_ and *H*_1_, respectively, are in the same topological phase if there is a local unitary that maps the two states into each other without closing the energy gap. In other words, there is a smooth path of local Hamiltonians *H*(*s*) that connects the two parent Hamiltonians with *H*(*s* = 0) = *H*_0_ and *H*(*s* = 1) = *H*_1_ for *s* ∈ [0, 1]. The Hamiltonian *H*(*s*) is gapped above its ground state *ρ*(*s*) for all *s* ∈ [0, 1], where *ρ*(*s*) smoothly connects *ρ*_0_ and *ρ*_1_, which establishes a smooth path between the two quantum many-body states. In ref. ^75^, it is show that such a path can be realized with a smoothly parametrized unitary *U*(*s*) whose generator *G*(*s*) is also a local operator, *ρ*(*s*) = *U*(*s*)*ρ*_0_*U*^†^(*s*), and naturally *ρ*_1_ = *U*(*s* = 1)*ρ*_0_*U*^†^(*s* = 1). This is further elaborated in Sec. II of the Supplementary Information.

The above Definition 2 is equivalent to the statement that the two topologically equivalent states can be deformed into each other by a constant-depth quantum circuit^4^, each layer of which can be divided into geometrically local quantum gate operations. Therefore, the two quantum states differ from each other only in entanglement patterns at short-range scales, while they are equivalent in their long-range entanglement structure.

Intuitively, the depth of the quantum circuit quantifies the scale of the change of the quantum entanglement that is caused by applying the circuit on the reference quantum state *ρ*_0_. Then, the quantum circuit complexity (QCC) for realizing *U*(*s* = 1) can serve as a measure of the algorithmic information for generating the target state *ρ*_1_ from a reference state *ρ*_0_. As is explained above, this is similar to, and inspired by, the (conditional) Kolmogorov complexity *K*(*x*∣*y*), which is the length of the shortest program on a universal computer that outputs a string or dataset *x* given the access of another string or dataset *y*, and as such represents an algorithmic information distance^59^ or the shared structure between the two objects. We adopt and adapt this here, using a geometric lower bound of the quantum circuit cost, Nielsen’s QCC ${{{{\mathcal{C}}}}}_{{{{\mathcal{N}}}}}\left({\rho }_{0}\to {\rho }_{1}\right)$, as a distance measure for clustering topological states with distinct entanglement patterns, whose definition is given in the following.

**Definition 3** (Nielsen’s QCC.) The quantum circuit complexity of the unitary *U*(*s* = 1) can be evaluated through Nielsen’s geometric approach^76,77^. For qubit systems, if we write *G*(*s*) = ∑_*σ *_*h*_*σ*_(*s*)*σ*, where *σ* ∈ *P*_*n*_ = {*I*, *X*, *Y*, *Z*}^⊗*n*^ and *σ* ≠ *I*^⊗*n*^ is a *n*-qubit Pauli operator (*I* is the 2-dimensional identity operator and *X*, *Y*, *Z* are the three single-qubit Pauli operators, respectively), and *h*_*σ*_(*s*) denote real coefficients, Nielsen’s quantum circuit complexity of the first order reads

2 $${{{{\mathcal{C}}}}}_{{{{\mathcal{N}}}}}\left({\rho }_{0}\to {\rho }_{1}\right)={\inf }_{h(s)}\,{\int }_{0}^{1}{\sum }_{\sigma }\left|{h}_{\sigma }(s)\right|\,{{{\rm{d}}}}s.$$

The original Nielsen’s QCC can be generalized to systems other than qubits^67,68,78^, where an orthonormal operator basis {*O*_*i*_} replaces the *n*-qubit Pauli operators *P*_*n*_, with the operator norm ∥*O*_*i*_∥_*∞*_≤1 (∀ *i*). Then the definition of Nielsen’s QCC above remains unchanged.

With Nielsen’s QCC of geometrically local quantum circuits as a topological and informational distance measure, the proposed theoretically optimal similarity/ kernel function for the unsupervised manifold learning of topological order is given by

3 $${{{\mathcal{K}}}}\left({\rho }_{0},{\rho }_{1}\right)=\exp \left[-\beta \,{{{{\mathcal{C}}}}}_{{{{\mathcal{N}}}}}\left({\rho }_{0}\to {\rho }_{1}\right)\right],$$

up to a normalization constant, where *β* > 0 is a hyperparameter. The unitarity of the quantum circuit indicates that Nielsen’s QCC is symmetric under permutations of the two quantum states, i.e., ${{{{\mathcal{C}}}}}_{{{{\mathcal{N}}}}}\left({\rho }_{0}\to {\rho }_{1}\right)={{{{\mathcal{C}}}}}_{{{{\mathcal{N}}}}}\left({\rho }_{1}\to {\rho }_{0}\right)$, which leads to a symmetric kernel in (3). The similarity function in (3) can be used to construct conditional probabilities in the diffusion map^51,52^ and the t-SNE^50^ manifold learning, or can be used for kernel PCA^53,54^ in the kernel feature space. Physically, Nielsen’s QCC can be viewed as a global order parameter of topological quantum orders, and also serves as an ideal topological distance in non-kernel manifold learning, such as the metric-MDS^55,56^.

### Unsupervised machine learning of topological order derived from quantum circuit complexity

While it is in general hard to exactly compute ${{{{\mathcal{C}}}}}_{{{{\mathcal{N}}}}}\left({\rho }_{0}\to {\rho }_{1}\right)$ for the QPP, we now establish some approximate substitutes, which both capture essential aspects of Nielsen’s QCC and at the same time are easier to implement in practice. The two theorems that we present in the following provide rigorous guarantees for this purpose.

First, we define the quantum Fisher complexity (QFC) related to the QPP, denoted by ${{{{\mathcal{C}}}}}_{{{{\mathcal{F}}}}}\left({\rho }_{0}\to {\rho }_{1}\right)$, which coincides with the Bures distance between the two quantum states given by a fidelity measure and can be efficiently calculated in practice.

**Definition 4 **[Quantum Fisher complexity (QFC).] Defining the Uhlmann-Jozsa fidelity between two arbitrary quantum states (pure or mixed) as given by (see ref. ^79^)

4 $$F\left(\rho,\widetilde{\rho }\right)={{{\rm{tr}}}}\sqrt{\sqrt{\rho }\widetilde{\rho }\sqrt{\rho }},$$

where ${{{\rm{tr}}}}$ denotes the trace operation, then the corresponding squared Bures distance reads^80–82^

5 $${D}_{B}^{2}\left(\rho,\widetilde{\rho }\right)=2\left[1-F\left(\rho,\widetilde{\rho }\right)\right].$$

For a family of quantum states *ρ*(*s*) smoothly parametrized by *s* ∈ [0,  1], the quantum Fisher information (QFI) of the quantum state with respect to the parameter *s* is given by (see refs. ^81–85^) ${{{{\mathcal{F}}}}}_{Q}\left(s\right)={{{\rm{tr}}}}\left[\rho \left(s\right){{{{\mathcal{L}}}}}^{2}\right]$, with the symmetric logarithmic derivative (SLD) ${{{\mathcal{L}}}}$ determined by ${\partial }_{s}\rho \left(s\right)=\frac{1}{2}\left[\rho \left(s\right){{{\mathcal{L}}}}+{{{\mathcal{L}}}}\rho \left(s\right)\right]$. The QFI, which plays a central role in quantum parameter estimation and which is also used for characterizing multipartite entanglement and topological states^86^, can be related to the infinitestmal Bures distance^80^ with

6 $$\frac{1}{4}{{{{\mathcal{F}}}}}_{Q}(s){{{{\rm{d}}}}}^{2}s={D}_{B}^{2}\left[\,\rho (s),\rho (s+{{{\rm{d}}}}s)\right],$$

representing a susceptibility of the fidelity. The QFC of the target quantum state *ρ*_1_ with respect to the reference state *ρ*_0_ is given by (see ref. ^66^)

7 $${{{{\mathcal{C}}}}}_{{{{\mathcal{F}}}}}\left({\rho }_{0}\to {\rho }_{1}\right)={\inf }_{h(s)}\,\frac{1}{2}{\int }_{0}^{1}\sqrt{{{{{\mathcal{F}}}}}_{Q}(s)}\,\,\,{{{\rm{d}}}}s,$$

which represents the optimal Bures distance between the two quantum states^82^, reflecting the overall fidelity variation, as indicated by (6). Note that we have a symmetric QFC under permutations of its inputs, i.e., ${{{{\mathcal{C}}}}}_{{{{\mathcal{F}}}}}\left({\rho }_{0}\to {\rho }_{1}\right)={{{{\mathcal{C}}}}}_{{{{\mathcal{F}}}}}\left({\rho }_{1}\to {\rho }_{0}\right)$.

With the QFC and the Bures distance, we are ready to formulate the following theorem.

#### Theorem 1

(Quantum circuit complexity upper bounds quantum Fisher complexity and Bures distance.) The QCC for the QPP from *ρ*_0_ to *ρ*_1_ is lower bounded by the QFC and the Bures distance,

8 $${{{{\mathcal{C}}}}}_{{{{\mathcal{N}}}}}\left({\rho }_{0}\to {\rho }_{1}\right) \ge \,	 {{{{\mathcal{C}}}}}_{{{{\mathcal{F}}}}}\left({\rho }_{0}\to {\rho }_{1}\right)\\ \ge 	 \frac{{D}_{B}\left({\rho }_{0},{\rho }_{1}\right)}{\sqrt{2}}.$$

Furthermore, the QCC of a geometrically local quantum circuit for the same QPP is approximately lower bounded by

9 $${{{{\mathcal{C}}}}}_{{{{\mathcal{N}}}}}\left({\rho }_{0}\to {\rho }_{1}\right)\gtrsim \frac{1}{\sqrt{2}}\,{\sum }_{\Delta }\,{D}_{B}\left[{\rho }_{0}\left(\Delta \right),{\rho }_{1}\left(\Delta \right)\right],$$

where $\rho \left(\Delta \right)$ is the reduced density matrix supported on the subsystem *Δ* of constant geometric size, and the summation goes over non-overlapping subsystems which, together with their neighboring environments, cover the whole system.

The proof of Theorem 1 is given in Sec. I of the Supplementary Information, and an asymptotic and tightness analysis of the bounds in Theorem 1 is provided in Sec. III. Note that, without the constraint of geometric locality of the quantum circuit, the overall Bures distance in (8) is equivalent to the *L*_2_-norm distance between the two density matrices, which has been shown not to be a suitable distance measure for topological phases^23^.

Under the geometric locality of the quantum circuit, we exploit Theorem 1 to construct a fidelity-based kernel. The Uhlmann-Jozsa fidelity between the *r*-body reduced density matrices with *r* = {1, 2, ⋯  , *R*} can be used for this purpose, where *R* is a scale of cutoff. For instance, for qubits hosted on a lattice Λ = [1, *L*]^*D*^ of lattice size *L* and dimension *D*, the proposed kernel based on fidelity reads (up to some normalization constant)

10 $${{{{\mathcal{K}}}}}_{{{{\rm{F}}}}}\left(\rho,\widetilde{\rho }\right)=\exp \left\{\beta {\sum }_{r=1}^{R}{\omega }_{r}{\sum }_{\Delta \in {P}_{r}(\Lambda )}F\left[\rho (\Delta ),\,\widetilde{\rho }(\Delta )\right]\right\},$$

where ${P}_{r}(\Lambda )=\{\Delta | \Delta \subset \Lambda \,\,{{{\rm{and}}}}\,\,\left|\Delta \right|=r\}$, with $\left|\Delta \right|$ being the size (cardinality) of the sublattice *Δ*; $\rho (\Delta )={{{{\rm{tr}}}}}_{(\Lambda -\Delta )}\rho$ is the reduced density matrix supported on *Δ*; *ω*_*r*_ is a weight function; and *F*(⋅ , ⋅) is the Uhlmann-Jozsa fidelity. While the dimension of the reduced density matrix depends exponentially on its size *r*, this fidelity-based kernel can be efficiently estimated in practical implementations for $R={{{\rm{const.}}}}$ (For instance, a small *r* = 2 is already sufficient in many relevant applications. See Theorem 1 and the section on numerical experiments below).

The second approximation of Nielsen’s QCC that we propose focuses on the entanglement change. Denoting by *ρ* a pure density matrix of a *n*-qubit chain (rearranged from an arbitrary spatial geometry following a specified order), and *A*_*k*_ = {1, 2, . . . , *k*} ⊂ [*n*] a subset of the system, which is obtained from a cut at *k* ∈ [1, *n* − 1], then the reduced density matrix supported on *A*_*k*_ reads $\rho ({A}_{k})={{{{\rm{tr}}}}}_{{B}_{k}}\rho$, where *B*_*k*_ = {*k* + 1, *k* + 2, . . . , *n*}. With the entanglement entropy over the cut *k* given by

11 $${S}_{k}(\,\rho )={{{\rm{tr}}}}\left[\rho ({A}_{k}){{{\rm{ln}}}}\,\rho ({A}_{k})\right],$$

we have the following theorem:

#### Theorem 2

[Quantum circuit complexity upper bounds entanglement-profile distance (A generalized version of Observation 1 in ref. ^78^)]. The QCC for the QPP from *ρ*_0_ to *ρ*_1_ with a geometrically local quantum circuit (with respect to the *n*-qubit chain) is lower bounded by

12 $${{{{\mathcal{C}}}}}_{{{{\mathcal{N}}}}}\left({\rho }_{0}\to {\rho }_{1}\right)\ge \frac{c}{\left(n-1\right)}{\sum }_{k=1}^{n-1}\left|{S}_{k}({\rho }_{1})-{S}_{k}({\rho }_{0})\right|,$$

for some constant *c* > 0.

The proof of Theorem 2 is given in Sec. I of the Supplementary Information, and an asymptotic and tightness analysis of the bounds in Theorem 2 is provided in Sec. III. Theorem 2 can be readily generalized to situations where the qubits are arranged in higher spatial dimensions and other geometries, provided that the quantum circuit is applied in a geometrically local manner accordingly. Nielsen’s QCC is then lower bounded by the entanglement change distributed over suitable cuts covering non-trivial bonds of the system.

Theorem 2 inspires an entanglement-based kernel, given by

13 $${{{{\mathcal{K}}}}}_{{{{\rm{E}}}}}\left(\rho,\widetilde{\rho }\right)=\exp \left(-\frac{\beta }{n}{\sum }_{k=1}^{n-1}\left|{S}_{k}(\rho )-{S}_{k}(\widetilde{\rho })\right|\right),$$

which is particularly useful when the entanglement profile over nontrivial bonds can be efficiently estimated. This is, for instance, the case in tensor-network simulations of quantum many-body states such as the density matrix renormalization group (DMRG)^87,88^, or in experimentally prepared quantum states measured via classical shadow tomography^89^.

Both lower bounds of Nielsen’s QCC in (9) and (12) potentially capture important information about the topological order. The fidelity-based kernel with reduced density matrices of constant size can be efficiently calculated and benchmarked (For instance, with classical shadow representations, see the following section). The entanglement-based kernel is more stringent and contains more information about quantum entanglement at multiple scales.

### Relation to classical shadows

Classical shadow (CS) tomography is an experimentally friendly approach to access many properties of quantum many-body states with few-shot measurements^89^. In this formalism, the quantum state is approximated by its CS estimator,

14 $$\rho \approx {S}_{T}\left(\rho \right)=\frac{1}{T}{\sum }_{t=1}^{T}{\sigma }_{1}^{(t)}\otimes {\sigma }_{2}^{(t)}\cdots \otimes {\sigma }_{n}^{(t)},$$

in a total of *T* measurements, where ${\sigma }_{i}^{(t)}$ is the local CS representation under the Pauli measurement for the *i*-th qubit in the *t*-th measurement. The shadow kernel suggested in ref. ^38^, given by

15 $${{{{\mathcal{K}}}}}_{{{{\rm{CS}}}}}\left[{S}_{T}\left(\rho \right),{S}_{T}\left(\widetilde{\rho }\right)\right]=\exp \left\{\frac{\beta }{{T}^{2}}{\sum }_{t,\,{t}^{{\prime} }=1}^{T}\,\exp \left[\frac{\nu }{n}{\sum }_{i=1}^{n}{{{\rm{tr}}}}\left({\sigma }_{i}^{(t)}{\widetilde{\sigma }}_{i}^{(t^{\prime})}\right)\right]\right\},$$

can be understood as a special case of the fidelity-based kernel in (10), if the quantum state *ρ* and the *r*-body reduced density matrices are represented by their classical shadows, if the weight function is taken to be *ω*_*r*_ = *ν*^*r*^/*r*!, and if we take the limit *R* → *∞*, where *β* > 0 and *ν* > 0 are constant hyperparameters. In this case, density matrices with small values of *r* dominate in the summation, and randomized measurements reduce the complexity in calculating fidelity-based kernels.

### Numerical experiments

In this section, we provide numerical demonstrations for the developed theory of unsupervised machine learning of topological order based on the QCC.

#### The bond-alternating XXZ spin chain

We begin with a benchmark model, the bond-alternating XXZ spin-$\frac{1}{2}$ chain^38,90,91^, whose Hamiltonian is given by

16 $${H}_{{{{\rm{XXZ}}}}}={\sum }_{i=1}^{n-1}{J}_{i}\left({X}_{i}{X}_{i+1}+{Y}_{i}{Y}_{i+1}+\delta {Z}_{i}{Z}_{i+1}\right)-{h}_{0}{\sum }_{i=1}^{n}{Z}_{i},$$

where *J*_*i*_ = *J*_1_ if *i* is odd and *J*_*i*_ = *J*_2_ if *i* is even, respectively; *δ* is a detuning parameter, and *h*_0_ denotes the strength of the external field.

**Fig. 2:**
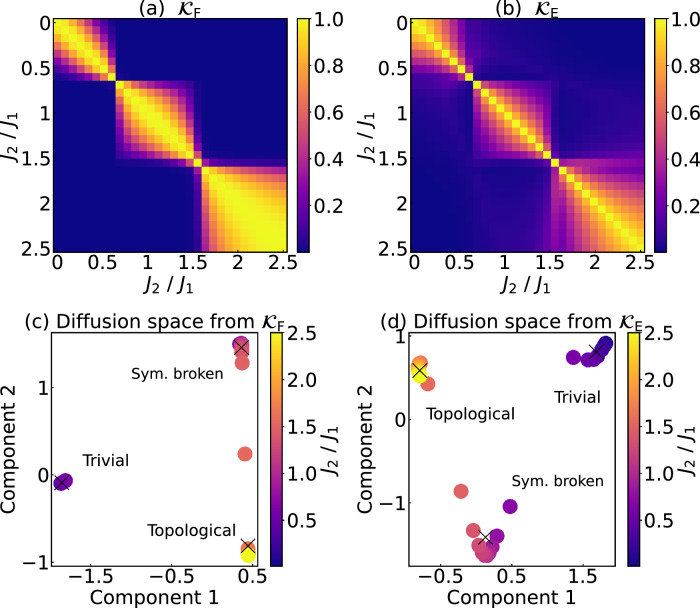
Unsupervised manifold learning of the bond-alternating XXZ qubit chain with fidelity- and entanglement-based informational distances. **a**, **b** show the fidelity- and entanglement-based kernels, ${{{{\mathcal{K}}}}}_{{{{\rm{F}}}}}\left(\rho,\widetilde{\rho }\right)$ and ${{{{\mathcal{K}}}}}_{{{{\rm{E}}}}}\left(\rho,\widetilde{\rho }\right)$, respectively. One clearly identifies three clusters in the heat map, corresponding to three distinct quantum phases of the model (trivial, symmetry (Sym.) broken, and topological) in a range of the model parameter *J*_2_/*J*_1_ ∈ (0, 2.5], which is represented by the two axes of (**a**) and (**b**), respectively. The colorbar encodes the value of the normalized kernel. **c**, **d** show the corresponding two-dimensional (2D) nonlinear representations of the samples, through the diffusion map algorithm [plotted are the second and the third dimensions in the diffusion space (Components 1 and 2, respectively), after normalization by the standard deviation]. The colorbar encodes the value of *J*_2_/*J*_1_, and the cross in each plot indicates the centre of the cluster found by a *k*-means clustering algorithm. The outlier in (**c**) with the value of *J*_1_/*J*_2_ ≈ 1.5 is the critical phase due to the finite-size effect and the resulting finite-width of the critical region. We use *n* = 151 qubits and the DMRG [with the singular value decomposition (SVD) cutoff 10^−10^ and the maximal energy error 10^−10^] to determine the ground state. We set *h*_0_ = 0 and *δ* = 3, while *N* = 30 samples are drawn uniformly in the parameter range of *J*_2_/*J*_1_ ∈ (0, 2.5] in an ordered manner. The hyperparameter of the kernel is *β* = 50.0 for (**a**) and (**c**); and *β* = 10.0 for (**b**) and (**d**). The entanglement profile is accessible in the DMRG calculation with the matrix product state representation. In the fidelity-based kernel, we use *n* geometrically local (nearest-neighbour) two-body reduced density matrices to cover the system, i.e., we only use terms with *r* = 2 and *ω*_*r*=2_ = 1/*n* in (10).

In Fig. 2, we compare the fidelity- and entanglement-based kernels for this model, together with the two-dimensional representation in the diffusion space from manifold learning of the diffusion map algorithm^22–24,51,52^. The ground-state properties (fidelity and entanglement) are obtained from DMRG calculations of the model. Fixing *h*_0_ = 0, *δ* = 3 and varying the value of *J*_2_/*J*_1_, we find that the unsupervised learning produces distinct clusters that can be associated with three phases (trivial, symmetry broken, and topological), in good agreement with independent calculations of topological invariants^38,90,91^. More details can be found in the caption of Fig. 2. While we are ultimately aiming at the unsupervised learning of topological order in the presence of non-local quantum entanglement, Fig. 2 shows that the fidelity- and entanglement-based kernels also perform well when clustering symmetry-broken phases and symmetry-protected topological orders^91^ that are characterized by short-range entanglement.

#### Kitaev’s toric code

As a second demonstration, we cluster the ground state of Kitaev’s toric code with random product states, where the former has topological quantum order while the latter are topologically trivial. The toric code Hamiltonian defined on a 2D lattice of size *L*_*x*_ × *L*_*y*_ is given by (see ref. ^7^)

17 $$\begin{array}{r}{H}_{{{{\rm{TC}}}}}=-{\sum }_{v}{A}_{v}-{\sum }_{p}{B}_{p},\end{array}$$

where *v* denotes a vertex and *p* is the face of the lattice, respectively; ${A}_{v}={\prod }_{i\in {{{\rm{star}}}}(v)}{X}_{j}$ and ${B}_{p}={\prod }_{i\in {{{\rm{boundary}}}}(p)}{Z}_{j}$ are the Kitaev’s star and face operators, respectively. The number of qubits for this model is *n* = 2*L*_*x*_*L*_*y*_. The toric code ground state is gapped and exhibits area-law entanglement^5^. Therefore, for the purpose of a proof-of-principle demonstration and in order to obtain the entanglement profile for the kernel with a small system size, we use an MPS representation and DMRG to find a reasonably accurate solution for the ground state. For instance, numerical experiments show that, for a 4 × 4 lattice with periodic boundary conditions in both dimensions, a maximal MPS bond dimension of 64 is sufficient to produce accurate results.

**Fig. 3:**
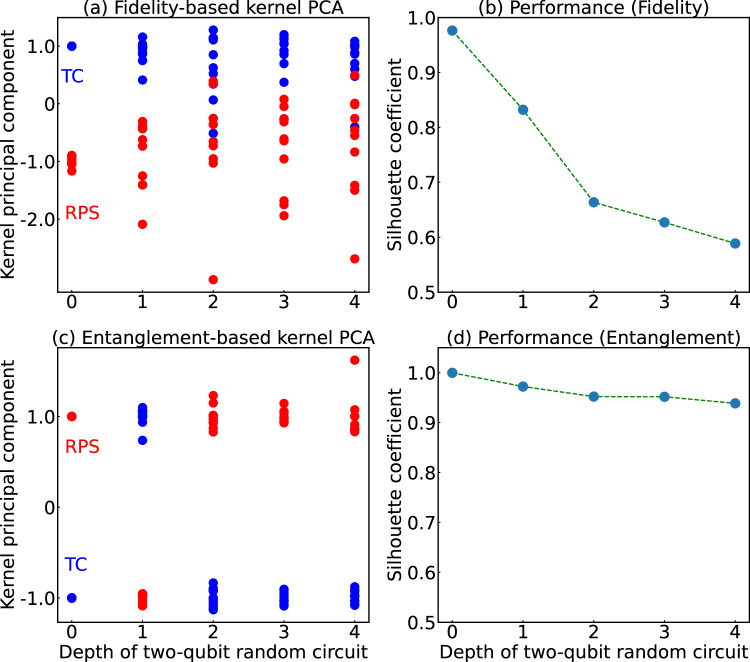
Unsupervised clustering of the ground state of Kitaev’s toric code (TC) [blue dots in sub-figures (a) and (c)] and random product states (RPS) [red dots in sub-figures (a) and (c)] without or with applying two-qubit random unitaries, via fidelity- and entanglement-based kernels, respectively. **a**, **c** show a one-dimensional (1D) representation of the dataset in terms of the kernel’s principal components (i.e., via the algorithm of kernel PCA with shifting the center to zero and normalizing by the standard deviation), by applying different depths of Haar random two-qubit unitaries^92^ repeatedly to both the toric code and the RPS (see the Methods seciton). **a**, **c** utilize the fidelity- and entanglement-based kernels, ${{{{\mathcal{K}}}}}_{{{{\rm{F}}}}}\left(\rho,\widetilde{\rho }\right)$ and ${{{{\mathcal{K}}}}}_{{{{\rm{E}}}}}\left(\rho,\widetilde{\rho }\right)$, respectively. **b**, **d** plot the degradation of clustering performance under Haar random unitaries for results in (**a**) and (**c**), respectively, with the silhouette coefficient^56^ (which takes the value one for best clustering) as a benchmark metric. The four subplots share the same horizontal axis. We use *N* = 20 samples in total, with 10 samples of the toric code (blue dots) and 10 samples of the RPS (random spin ups and downs of *n* qubits) (red dots). We use *n* = 32 qubits (on a lattice of size 4 × 4 with toric boundary condtions; i.e., with a small code distance of 4) for this proof-of-principle demonstration, and the DMRG (with the SVD cutoff 10^−10^ and the maximal energy error 10^−10^) to solve for the ground state of the toric code and, at the same time, to extract the entanglement profile. The hyperparameter of the kernel is *β* = 0.1 for (**a**) and *β* = 2.0 for (**c**). With the fidelity-based kernel, we randomly sample *n* two-body reduced density matrices, i.e., we only use terms with *r* = 2 and *ω*_*r*=2_ = 1/*n* in (10). The results show that, in the absence of random unitaries (i.e., with circuit depth zero), both kernel types perform well in clustering the toric code and the RPS, while the entanglement-based kernel in (**c**) is found to be more robust in the presence of Haar random two-qubit unitaries [see (**b**, **d**)].

In Fig. 3 we plot a comparison between the unsupervised machine learning of the toric code ground state (blue dots) and the random product states (RPS) (red dots) of qubits (i.e., random bit strings of spin ups and downs), with fidelity- and entanglement-based kernels, respectively. Both kernels perform well in clustering the topologically ordered quantum state and the trivial phase, while the entanglement-based one is more robust under the application of two-qubit Haar random unitaries to both the toric code and the RPS. This follows from the comparison between Fig. 3b, d, where the silhouette coefficient^56^, which takes the value one for best clustering, is used for illustrating the performance degradation of the clustering results in Fig. 3a, c, respectively, under Haar random unitaries. Here, we do not incorporate geometric locality for the two-body reduced density matrices in calculating the fidelity-based kernel (as not required explicityly in the shadow kernel learning). Moreover, we find that, with geometric locality, the clustering performance of the fidelity kernel PCA will be enhanced, but is still inferior to that of the entanglement-based one under Haar random unitaries. More details about the plot can be found in the caption of Fig. 3.

For more discussions on the effects of hardware noises and applied random quantum circuits, we refer the reader to Sec. V of the Supplementary Information. In addition, in Sec. VI of the Supplementary Information (see Supplementary Fig. S1), we also use metric-MDS (which does not require a kernel formulation) based on the Bures and entanglement-profile distance metrics in (9) and (12), respectively, for unsupervised manifold clustering of the toric code and the product state. Similar conclusions can be obtained from Supplementary Fig. S1.

#### The extended toric code

The original toric code is defined on a torus with periodic boundary conditions in both spatial dimensions. In contrast, the extended toric code (ETC) is defined on an infinite cylinder (e.g., the cylinder axis is along the *x* direction and the *y* direction is periodic with circumference *L*_*y*_), and additionally features the Wilson and t’Hooft loop operators *W* and *H*, respectively. The corresponding Hamiltonian is given by (see ref. ^92^)

18 $${H}_{{{{\rm{ETC}}}}}={H}_{{{{\rm{TC}}}}}-{J}_{{{{\rm{W}}}}}W-{J}_{{{{\rm{H}}}}}H-h{\sum }_{i=1}^{n}{Z}_{i},$$

where *H*_TC_ is the original toric code Hamiltonian in (17), and *h* is a (uniform) local field strength. The loop operators are defined as products

19 $$W={\prod }_{i\in {E}_{v}}{Z}_{i},\,\,\,\,\,H={\prod }_{i\in {E}_{h}}{X}_{i}$$

around the cylinder through vertical edges (*E*_*v*_) and horizontal edges (*E*_*h*_) of the lattice graph, respectively.

**Fig. 4:**
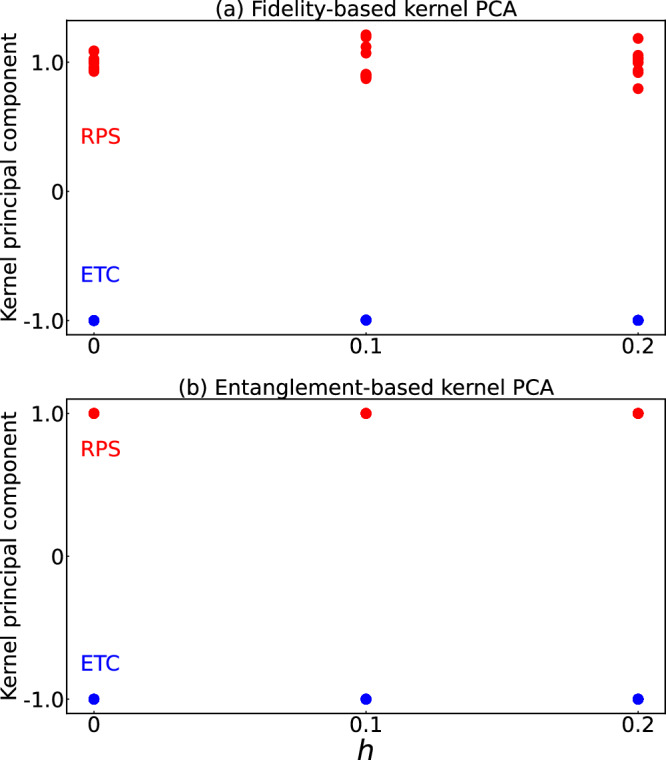
Unsupervised clustering of the ground state of the extended toric code (ETC) (blue dots) and random product states (RPS) (red dots) without or with applying local perturbations, via fidelity- and entanglement-based kernels, respectively. **a**, **b** show a one-dimensional (1D) representation of the dataset in terms of the kernel’s principal components (i.e., via the algorithm of kernel PCA with shifting the center to zero and normalizing by the standard deviation), by applying different field strengths of local perturbations (*h*) to the ETC. **a**, **b** utilize the fidelity- and entanglement-based kernels, ${{{{\mathcal{K}}}}}_{{{{\rm{F}}}}}\left(\rho,\widetilde{\rho }\right)$ and ${{{{\mathcal{K}}}}}_{{{{\rm{E}}}}}\left(\rho,\widetilde{\rho }\right)$, respectively. The two subplots share the same horizontal axis. We use *N* = 20 samples in total, with 10 samples of the ETC (blue dots) and 10 samples of the RPS (random spin-ups and downs of *n* qubits) (red dots). The boundary condition  is given by an infinite cylinder geometry, where it is periodic in the *y* direction with a circumference of *L*_*y*_ = 4; In the *x*-direction it is infinite, and we choose a unit cell of width *L*_*x*_ = 2. The MPS-based DMRG (with the SVD cutoff 10^−10^ and the maximal energy error 10^−10^) is used to solve for the ground state of the ETC and, at the same time, to extract the entanglement profile for both zero and nonzero values of *h*. The hyperparameter of the kernel is *β* = 0.2 for (**a**) and *β* = 2.0 for (**b**). With the fidelity-based kernel, we randomly sample *n* two-body reduced density matrices (as in Fig. 3), i.e., we only use terms with *r* = 2 and *ω*_*r*=2_ = 1/*n* in (10). The results show that the topological clustering is robust to local perturbations.

In Fig. 4, by fixing the values of *J*_*W*_ = + 1 and *J*_*H*_ = − 1, which stabilizes the ground state into one of the four degenerate sectors, and by varying the strength of the local perturbation field *h*, we plot a comparison between the unsupervised machine learning of the ETC ground state (blue dots) and the random product states (RPS) (red dots) of qubits (i.e., random bit strings of spin ups and downs), with fidelity- and entanglement-based kernels, respectively. Note that, for finite values of *h*, the ground states are no longer exactly solvable. The required maximal MPS bond dimension increases rapidly with larger values of *h* even for small system sizes (For the system size and the parameter range we adopted in Fig. 4, a maximal bond dimension of 600 is sufficient to produce accurate results). The topological clustering shows its stability against local perturbations. More details about the plot can be found in the caption of Fig. 4.

## Discussion

In summary, we used quantum circuit complexity, a vital concept in quantum computation, to understand and to build viable distance measures and kernels for the unsupervised machine learning of topological order, targeting interpretability and generalizability. By resorting to Nielsen’s quantum circuit complexity, two easier-to-implement distance metrics based on fidelity and entanglement were proposed, and their effectiveness numerically verified. Equipped with our topologically informational distance metrics, both kernel-based and non-kernel manifold learning can be applied for interpretable phase clustering of quantum many-body systems.

The shadow kernel learning of ref. ^38^ can be naturally explained by our method derived from Nielsen’s QCC, when the fidelity-based distance and classical shadow representation of the quantum state are used. The alternative approach based on the entanglement-entropy profile encodes more information about entanglement patterns in topological orders, and thus exhibits superior robustness against two-qubit random noises; moreover, they are more interpretable in terms of the relationship between topological quantum orders and long-range entanglement. Recent theoretical studies^93,94^ show the promise of efficient measurement of many-body entropies, which indicates that the entanglement-based distance can be calculated efficiently from experimental data. This implies that our method can be readily applied to experimentally prepared multi-qubit quantum states; for instance, data obtained from near-term quantum computing hardware. When limited to SPT order and compared to the recent results in ref. ^95^ about neural-network learning topological orders of the bound-alternating XXZ model, our method is fully unsupervised and results in sharper phase boundaries in clustering quantum phases of this model, while providing good interpretability.

In future work, the kernels proposed here may also be used for supervised learning tasks, such as classification or regression of topological phases, with an ${{{\mathcal{O}}}}(1)$ sample complexity. The nonlinear feature vectors (e.g., random Fourier features) given by the entanglement kernel can be used to learn about properties related to entanglement structures of the quantum many-body state. Also, in the demonstrated examples, so far we focused on pure and gapped ground states. Possible extensions could be applications in gapless, topological quantum criticalities, e.g., with the multiscale entanglement renormalization ansatz (MERA)^96^, or in the unsupervised machine learning of entanglement transitions^97,98^, as well as mixed-state topological order^99–103^.

Our current approach is primarily based on the classical machine learning of topological quantum order by exploiting Nielsen’s QCC as an informational distance. It might also be interesting to explore quantum machine learning^104,105^ in terms of parametrized quantum circuits (e.g., ref. ^106^), which may find approximately the minimal quantum circuit matching the reference and target quantum states in the dataset. The combination with reinforcement learning for quantum state generation^107–109^ is also promising. We also note a very recent work^110^ which uses exact quantum algorithms for topological quantum phase recognition, while restricted to one-dimensional SPT phases.

We believe that the results presented here make, both conceptually and technically, an important step toward an interpretable and generalizable theory of unsupervised machine learning of topological order, and as such will boost the interplay between the research fields of (interpretable) machine learning phases of matter, quantum complexity, quantum parameter estimation, and quantum computation.

## Methods

### Theoretical framework and proof ideas of Theorems 1 and 2

The core of our approach is an informational distance metric for topological dissimilarities, leveraging Nielsen’s geometric QCC of mapping two quantum many-body states as a primitive distance metric for manifold learning. This can result in both good interpretability and generalizability for unsupervised machine learning of topological quantum order. The theoretical toolbox includes differential geometry, quantum Fisher information, entanglement-growth rate, the concept of topological equivalence, and quantum circuit computation. By bridging conceptual power to practical implementation, Theorems 1 and 2 lead to approximate substitutes of the QCC, i.e., the Bures distance (fidelity change) and the entanglement-entropy distance, respectively, which preserve partial but important information of topological dissimilarity and which are intuitively more comprehensive and more accessible in simulations and measurements.

Now we describe the key ideas behind the proofs of Theorems 1 and 2. The proof of Theorem 1 relies on the insight  that the integrand of the first-order Nielsen’s QCC (time complexity of the QPP) is lower bounded by the operator norm of the generator of the QPP (easy to obtain via the Pauli-string-operator basis), which also upper bounds the QFI (or the Fubini-Study metric) during the QPP. The constraint of geometrically local generators can be naturally incorporated via the Trotterization of the QPP into very small time steps [see details of the proof in Sec. I of the Supplementary Information (SI)]. In the proof of Theorem 2, one finds that the result for the Small Incremental Entangling (SIE)^111–115^ is applicable to general QPPs, generalizing the initial product state^78^ to an arbitrary quantum many-body state. This is useful in our manifold learning of arbitrary quantum states, the distance between which is a key ingredient in the method. Similarly, geometrically local generators and Trotterization are used in the proof of Theorem 2 (see details of the proof in Sec. I of the SI).

Note that Nielsen’s QCC, in general, does not require an adiabatic (gapped) QPP. However, assuming a gapped path (ground state) allows us to estimate the asymptotic scaling (with respect to the number of qubits *n*) of Nielsen’s QCC and the quantum Fisher complexity (see Secs. II and III in the SI for details; and also for a tightness analysis of the derived bounds in the two theorems of the main text).

### Machine learning algorithms and numerical experiments

The DMRG ground-state search in the numerical experiments was performed using the TeNPy Library^92^, and some example codes there are reused for preparing the dataset of our machine learning model. In order to simulate the effects of random circuits or noise on the clustering performance of the method (see Fig. 3 and Supplementary Fig. S1), random unitary evolutions are applied to both the toric code and product states, via the time-evolving block decimation (TEBD) in the TeNPy Library^92^. This performs local two-site Haar random unitaries by a variable number of steps (two-qubit circuit depths). To implement the code in a reansonable time and memory, a maximum TEBD bond dimension can be set (e.g., to 500), and such random unitary evolutions of different circuit depths can be applied repeatedly to reach the maximum target bond dimension. More discussions on the effects of noise and random circuits can be found in Sec. V of the SI.

Regarding specific manifold-learning algorithms for dimensionality reduction and phase clustering, we use the well-established framework with diffusion map^23,51,52^, kernel PCA^53,54^, and metric-MDS^55,56^ for demonstrations in the current work. We expect  that other manifold-learning algorithms, such as the t-SNE^50^ and the Isomap^48^, equipped with our topologically informational distances, will give similar results.

In Sec. IV of the SI, we give an elaborated analysis on the sample and computational complexities of the method in terms of the number of qubits *n* and the sample size *N*, which shows that provably efficient unsupervised learning derived from Nielsen’s QCC is accessible in simulations and experiments. In addition, a (non-kernel and iterative) metric-MDS manifold learning [with a computational time of ${{{\mathcal{O}}}}\left({N}^{2}\right)$] of the toric code is also provided in Sec. VI of the SI, which is more efficient compared to kernel approaches [with a computational time of ${{{\mathcal{O}}}}({N}^{3})$] in case of large datasets.

## Supplementary information

Supplementary Information

Transparent Peer Review file

## Data Availability

All the data that support the findings of this study have been deposited in the Figshare^116^. All the data that support the findings of this study are also available from the corresponding authors upon request.

## Code availability

Source code for generating all the data and for plotting Figs. 2–4 in the main text and Supplementary Fig. S1 in the SI is available at Figshare^116^, and is also available at the Code Ocean capsule^117^ linked to this article.
