# Supplementary material for: Quantum circuit complexity and unsupervised machine learning of topological order
Source: Nat Commun. 2026 Apr 8;17:5179. doi: 10.1038/s41467-026-71283-5 (PMC13254324; doi:10.1038/s41467-026-71283-5)
Supplement: Supplementary file 1 — Supplementary Information [file 41467_2026_71283_MOESM1_ESM.pdf]

SUPPLEMENTARY INFORMATION for

"Quantum circuit complexity and unsupervised machine learning of topological order"

Yanming Che,[1, 2] Clemens Gneiting,[2] Xiaoguang Wang,[3] and Franco Nori[1, 2]

[1]*Department of Physics, University of Michigan, Ann Arbor, Michigan 48109-1040, USA*

[2]*Center for Quantum Computing, RIKEN, Wako-shi, Saitama 351-0198, Japan*

[3] *Zhejiang Key Laboratory of Quantum State Control and Optical Field Manipulation, Department of Physics, Zhejiang Sci-Tech University, Hangzhou 310018, China*

Table of contents

I. Proof of Theorems 1

II. Topological equivalence of gapped ground states and quantum circuit complexity of adiabatic quantum computation 4

III. Asymptotic and tightness analysis of the bounds in Theorems 1 and 2 5

IV. Complexity and scaling analysis of the method 6

V. Effects of noise and random quantum circuits 7

VI. Manifold learning of the toric code based on the metric-multidimensional scaling (metric-MDS) 8

I. Proof of Theorems

Proof of Theorem 1: For a pure quantum state $\rho(s) = |\psi(s)\rangle\langle\psi(s)|$ in the unitary evolution defined in the QPP of the main text, the QFI for the parameter s reads

$$\begin{aligned}\mathcal{F}_Q(s) &= 4\left(\langle\partial_s\psi(s)|\partial_s\psi(s)\rangle - |\langle\partial_s\psi(s)|\psi(s)\rangle|^2\right)\\ &= 4\left\{\mathrm{tr}\left(G^2(s)\rho(s)\right) - \left[\mathrm{tr}\left(G(s)\rho(s)\right)\right]^2\right\}\\ &= 4\mathrm{Var}\left[G(s)\right],\end{aligned} \tag{S1}$$

where we have used the relation $|\psi(s)\rangle = U(s)|\psi(0)\rangle$ and the hermitian generator $G(s) = i\left[\partial_s U(s)\right] U^\dagger(s)$; and Var denotes the variance with respect to the *evolved* quantum state $\rho(s)$. The variance of the generator $G(s)$ is upper bounded by

$$\mathrm{Var}\left[G(s)\right] \leq \frac{1}{4}\left[\lambda_{\max}(s) - \lambda_{\min}(s)\right]^2 \leq \|G(s)\|_\infty^2, \tag{S2}$$

where $\lambda(s)$ is the eigenvalue of $G(s)$; and to obtain the last inequality, we have used $|a-b| \leq |a| + |b|$ for real numbers a and b, and that, by definition, the operator norm of the generator $\|G(s)\|_\infty = |\lambda(s)|_{\max}$. The first inequality in (S2) is saturated when

$|\psi(s)\rangle = \frac{1}{\sqrt{2}}\left(|\lambda_{\text{max}}(s)\rangle + |\lambda_{\text{min}}(s)\rangle\right)$, i.e., the quantum state is an equal weight superposition of eigenstates with maximal and minimum eigenvalues of $G(s)$, respectively; and the last inequality is saturated when $\lambda_{\text{max}}(s) = -\lambda_{\text{min}}(s) > 0$.

On the other hand, the operator norm of the $G(s) = \sum_\sigma h_\sigma(s)\,\sigma$ of Nielsen's circuit complexity is upper bounded by

$$\|G(s)\|_\infty \leq \sum_\sigma |h_\sigma(s)|, \tag{S3}$$

where we have used the subadditivity of the operator norm and $\|\sigma\|_\infty = 1$ for n-qubit Pauli operators σ. Therefore, we have

$$\sqrt{\mathcal{F}_Q(s)} \leq 2\sum_\sigma |h_\sigma(s)| \tag{S4}$$

for *all possible paths* $h_\sigma(s)$ ($s \in [0,1]$). Then by integrating over $s \in [0,1]$ on both sides of (S4) followed by taking the infimum over all possible paths, the first inequality in (8) of the main text holds naturally, i.e.,

$$\mathcal{C}_{\mathcal{F}}\left(\rho_0 \to \rho_1\right) \leq \mathcal{C}_{\mathcal{N}}\left(\rho_0 \to \rho_1\right). \tag{S5}$$

The second inequality in (8) of the main text,

$$\frac{D_B\left(\rho_0, \rho_1\right)}{\sqrt{2}} \leq \mathcal{C}_{\mathcal{F}}\left(\rho_0 \to \rho_1\right), \tag{S6}$$

which lower bounds the QFC by the Bures distance between ρ_0 and ρ_1, can be readily and easily derived from the result in [S1].

The above proof can be directly generalized to systems other than qubits. Suppose that the generator of the unitary quantum path planning is

$$G(s) = \sum_i h_i(s)\, O_i, \tag{S7}$$

where O_i is an orthonormal operator basis with operator norm $\|O_i\|_\infty \leq 1$, then, by replacing the summation in the right-hand side of (S4) to go over all orthonormal operator bases of the generator, (S4) also holds, i.e.,

$$\sqrt{\mathcal{F}_Q(s)} \leq 2\sum_i |h_i(s)|. \tag{S8}$$

Next, a suitable similarity measure for topological quantum order imposes the additional constraint of geometric locality of the QPP, which requires that O_i is a geometrically local operator for all possible values of i, i.e., O_i is supported on a constant number of neighboring particles. We consider a reduced density matrix $\rho\left(s|\Delta\right)$ supported on a subsystem Δ of constant size, undergoing a nonunitary evolution along the unitary QPP of the total system generated by $G(s)$. While the relation between the Bures distance and the QFI in (6) of the main text still holds for mixed quantum states with the Uhlmann-Jozsa fidelity, the expression of the QFI for $\rho\left(s|\Delta\right)$ with respect to s is more complicated than (S1). Now the QFI has the general form given by the symmetric logarithmic derivative (SLD) $\mathcal{L}$, with

$$\mathcal{F}_Q\left(s|\Delta\right) = \operatorname{tr}\left[\rho\left(s|\Delta\right)\mathcal{L}^2\right], \tag{S9}$$

where $\mathcal{L}$ is determined by

$$\partial_s \rho\left(s|\Delta\right) = \frac{1}{2}\left[\rho\left(s|\Delta\right)\mathcal{L} + \mathcal{L}\rho\left(s|\Delta\right)\right]. \tag{S10}$$

We make a Trotter decomposition of the unitary evolution generated by $G(s)$ into a product of many sufficiently small, finite time steps. At each Trotter time step, we can estimate an upper bound for $\mathcal{F}_Q\left(s|\Delta\right)$ of the subsystem. The evolution of the

reduced density matrix of the subsystem Δ results from a partial-trace operation (with respect to the rest of the system) on the total system which undergoes the unitary evolution generated by $G(s)$. The QFI of the reduced density matrix with respect to s does not exceed that of its enlarged pure system, i.e.,

$$\mathcal{F}_Q\left(s|\Delta\right) \leq \mathcal{F}_Q\left(s\right), \tag{S11}$$

where the latter can be estimated via (S8) through unitary evolution. Note that (S11) can be readily obtained with the monotonicity of the Uhlmann-Jozsa fidelity of two quantum states ρ and $\tilde{\rho}$ [S2], i.e.,

$$F\left[\mathcal{E}(\rho),\ \mathcal{E}(\tilde{\rho})\right] \geq F\left(\rho,\ \tilde{\rho}\right) \tag{S12}$$

under the trace-preserving quantum operation $\mathcal{E}$, which means that the Bures distance and QFI are contractive under the partial-trace operation.

Due to the geometric locality of the generator and of the quantum circuit applied to the system as well as the *very small evolved time step*, the generators $\{O_i\}$ that govern the time evolution of the subsystem Δ are restricted to include only those supported on Δ and its neighboring environment, where the latter is directly coupled to Δ by applying the quantum circuit. We denote $\tilde{\Delta}$ as the subsystem Δ and its neighboring environment, where the size $|\tilde{\Delta}| \leq$ constant. Consequently, the summation on the right-hand side of (S8) for upper bounding $\mathcal{F}_Q\left(s|\Delta\right)$ must be restricted to the basis operators which are locally supported on Δ and its neighboring environment, i.e.,

$$\frac{1}{2}\sqrt{\mathcal{F}_Q\left(s|\Delta\right)} \leq \frac{1}{2}\sqrt{\mathcal{F}_Q\left(s\right)} \leq \sum_{i\,:\,\mathcal{S}_i\subset\tilde{\Delta}} |h_i(s)|, \tag{S13}$$

where $\mathcal{S}_i$ is the support (on the system) of the i-th orthonormal basis operator O_i in the expansion of the generator $G(s)$.

Then, by integrating over $s \in [0,\ 1]$, followed by summing over different such subsystems of constant size to cover the whole system in order to cover the support of the generator, we have

$$\sum_{\Delta} \frac{1}{2}\int_0^1 \sqrt{\mathcal{F}_Q\left(s|\Delta\right)}\,\mathrm{d}s \leq \int_0^1 \sum_{\Delta} \sum_{i\,:\,\mathcal{S}_i\subset\tilde{\Delta}} |h_i(s)|\,\mathrm{d}s \approx \int_0^1 \sum_i |h_i(s)|\,\mathrm{d}s, \tag{S14}$$

where the summation in the right-hand side of the last approximate equation goes over all operator bases of the generator. Moreover, from the result presented in [S1], we can derive that

$$\frac{1}{\sqrt{2}} D_B\left[\rho_0\left(\Delta\right), \rho_1\left(\Delta\right)\right] \leq \frac{1}{2}\int_0^1 \sqrt{\mathcal{F}_Q\left(s|\Delta\right)}\,\mathrm{d}s, \tag{S15}$$

which always holds for arbitrary smooth paths between the two reduced density matrices, caused by the smooth unitary path of the total system followed by the partial-trace operation, i.e., it is path-independent. Then we can take the infimum over all possible paths of the unitary QPP on the right-hand side of the last equation in (S14) to obtain a tighter bound, and conclude that Nielsen's QCC of geometrically local quantum circuits is approximately lower bounded by the summation over fidelity distances of the reduced density matrices supported on non-overlapping subsystems $\{\Delta\}$ which, together with their neighboring environments, cover the total system, i.e.,

$$\mathcal{C}_{\mathcal{N}}\left(\rho_0 \to \rho_1\right) \gtrsim \frac{1}{\sqrt{2}} \sum_{\Delta} D_B\left[\rho_0\left(\Delta\right), \rho_1\left(\Delta\right)\right]. \tag{S16}$$

(This completes the proof of Theorem 1.)

Proof of Theorem 2: Here we follow a similar procedure as in [S3], but with more details in order to generalize the result to two arbitrary multi-qubit states in a generic QPP problem. Arrange the n-qubit system into a qubit chain $\{1, 2, \cdots, n\}$ following

a specific order, and take a subsystem over the k-th cut, $A_k = \{1, 2, \cdots, k\}$, and its environment $B_k = \{k+1, k+2, \cdots, n\}$ as in the statement before Theorem 2. Further, we assume that the coupling in the generator $G(s)$ of $U(s)$ between A_k and B_k is spatially local with respect to the qubit chain. By Trotter decomposition, slicing $s \in [0,\, 1]$ into small time steps $\{s_j\}_{j=0}^{T_s-1}$, and at each time interval $\Delta s_j = |s_j - s_{j-1}|$ ($j \in [1, T_s - 1]$), the unitary evolution operator of the QPP is given by

$$U(s_j) = \exp\left[-i \Delta s_j G(s_j)\right]. \tag{S17}$$

The generators $G(s_j)$ can be decomposed as

$$G(s_j) = G_{A_k}(s_j) + G_{B_k}(s_j) + G_k(s_j), \tag{S18}$$

where $G_{A_k}(s_j)$ and $G_{B_k}(s_j)$ are supported on A_k and B_k, respectively, and

$$G_k(s_j) = \sum_{O^{(k)}} h_{O^{(k)}}\left(s_j\right) O^{(k)}, \tag{S19}$$

is the local coupling between the subsystems A_k and B_k, with $O^{(k)}$ a geometrically local basis operator at the k-th bond of operator norm $\left\|O^{(k)}\right\|_\infty \le 1$. With rigorous results already proved in [S4–S8] concerning the *Small Incremental Entangling (SIE)*, the rate of the entanglement over the cut k generated or destroyed in this small time slice is upper bounded by

$$\begin{aligned}\left|\frac{\mathrm{d}}{\mathrm{ds}} S_k\left[\rho(s)\right]\right|\Bigg|_{s=s_j} &\le c' \,\ln d_k\, \left\|G_k(s_j)\right\|_\infty \\ &\le c' \,\ln d_k \sum_{O^{(k)}} \left|h_{O^{(k)}}\left(s_j\right)\right|,\end{aligned} \tag{S20}$$

where $c' > 0$ is a constant and $d_k = \min\{d_{A_k}, d_{B_k}\} \le 2^{n-1}$ is the minimal Hilbert-space dimension of the subsystems. With (S20), integrating over $s \in [0,\, 1]$ (summing over all infinitestmal time slices Δs_j) and using the relation $\left|\int_0^1 f(s) \mathrm{d}s\right| \le \int_0^1 |f(s)|\, \mathrm{d}s$ for smooth functions, followed by summing over all cuts k, we obtain that Nielsen's circuit complexity measure is, for all possible paths, lower bounded by the entanglement change averaged over the bonds. We can then take the infimum of the circuit complexity measure over all paths allowed to obtain a tighter bound, i.e.,

$$\frac{c}{(n-1)} \sum_{k=1}^{n-1} \left|S_k(\rho_1) - S_k(\rho_0)\right| \le \mathcal{C}_{\mathcal{N}}\left(\rho_0 \to \rho_1\right), \tag{S21}$$

for some constant $c = 1/\left(c' \ln 2\right) > 0$. Note that here the reference quantum state ρ_0 is not restricted to a product state, and we find an averaging prefactor $1/(n-1)$ compared to the result in [S3]. (This completes the proof of Theorem 2.)

II. Topological equivalence of gapped ground states and quantum circuit complexity of adiabatic quantum computation

As mentioned in Definition 2, two gapped quantum states ρ_0 and ρ_1 in the same topological phase can be connected via a smooth path $\rho(s) = U(s)\rho_0 U^\dagger(s)$ for $s \in [0, 1]$. In case of gapped spectra, an explicit expression for the generator $G(s)$ is provided in [S9], within the spectral flow formalism:

$$G(s) = \int_{-\infty}^{+\infty} \mathrm{d}t\; W_\gamma(t)\, \mathrm{e}^{itH(s)}\, \partial_s H(s)\, \mathrm{e}^{-itH(s)}, \tag{S22}$$

where $H(s)$ is the Hamiltonian path with a spectral gap $\delta(s) \ge \gamma > 0\, (\forall s \in [0, 1])$ and with the ground state $\rho(s)$; $W_\gamma(t) \in L^1(\mathbb{R})$ is a decaying weight function of the spectral flow satisfying $\left\|W_\gamma\right\|_1 \le c_0/\gamma$, for some positive constant c_0. In this

situation, the Nielsen QCC may be explicitly obtained by optimization based on differential geometry, which deserves further investigation.

From the Proof of Theorem 1, we know that the integrand of Nielsen's QCC is lower bounded by the operator norm of the generator, with $\|G(s)\|_\infty \leq \sum_\sigma |h_\sigma(s)|$. This inequality is already sufficiently satisfied if we replace $\|G(s)\|_\infty$ on the left-hand side by its maximal value. With (S22) and the properties of the decaying weight function of the spectral flow, one finds the maximal value to satisfy

$$\|G(s)\|_\infty \leq \frac{c_0\|\partial_s H(s)\|_\infty}{\gamma}. \tag{S23}$$

It follows that Nielsen's QCC can be estimated as

$$\mathcal{C}_\mathcal{N} \sim \mathcal{O}\left(\max_{s\in[0,1]} \frac{\|\partial_s H(s)\|_\infty}{\gamma}\right), \tag{S24}$$

which is similar (but not identical) to the adiabaticity condition in adiabatic quantum computation [S10].

III. Asymptotic and tightness analysis of the bounds in Theorems 1 and 2

For gapped ground states, we will show in the following that the quantum Fisher complexity (QFC) in Theorem 1 is asymptotically (with respect to the number of qubits n) tight as a lower bound of the Nielsen's QCC. For the quantum path planning following the gapped ground state $\rho(s)$ of the Hamiltonian $H(s)$, one can write the quantum Fisher information of the ground state $\rho(s) = |\psi_0(s)\rangle\langle\psi_0(s)|$ as [S11, S12]

$$\mathcal{F}_Q(s) = 4\sum_{m\neq 0} \frac{|\langle\psi_m(s)|\partial_s H(s)|\psi_0(s)\rangle|^2}{[E_m(s) - E_0(s)]^2}, \tag{S25}$$

where the Hamiltonian has a spectral representation $H(s) = \sum_{m=0} E_m(s)|\psi_m(s)\rangle\langle\psi_m(s)|$ with $E_m(s) \leq E_{m+1}(s)$ for $m = 0, 1, 2, \cdots$, and a spectral gap $E_1(s) - E_0(s) \geq \gamma > 0\,(\forall s \in [0, 1])$. Note that the spectral gap γ can scale as $1/\text{poly}(n)$ or $1/\exp(n)$.

With (S25), an upper bound for the quantum Fisher information can be readily obtained,

$$\begin{aligned}
\mathcal{F}_Q(s) &\leq \frac{4}{\gamma^2}\sum_{m\neq 0} \langle\psi_0(s)|\partial_s H(s)|\psi_m(s)\rangle\langle\psi_m(s)|\partial_s H(s)|\psi_0(s)\rangle \\
&= \frac{4}{\gamma^2}\langle\psi_0(s)|\partial_s H(s)\left(\mathcal{I} - |\psi_0(s)\rangle\langle\psi_0(s)|\right)\partial_s H(s)|\psi_0(s)\rangle \\
&= \frac{4}{\gamma^2}\text{Var}_{|\psi_0(s)\rangle}[\partial_s H(s)] \\
&\leq \frac{4}{\gamma^2}\|\partial_s H(s)\|_\infty^2,
\end{aligned}$$

where $\mathcal{I} = \sum_{m=0} |\psi_m(s)\rangle\langle\psi_m(s)|$ is the identity, and we have used (S2) to obtain the last inequality.

Therefore, the QFC is

$$\mathcal{C}_\mathcal{F} \sim \mathcal{O}\left(\max_{s\in[0,1]} \frac{\|\partial_s H(s)\|_\infty}{\gamma}\right), \tag{S26}$$

which is asymptotically same as Nielsen's QCC $\mathcal{C}_\mathcal{N}$ in (S24). The scaling with respect to the system size is dominated by the spectral gap $\gamma \sim 1/\text{poly}(n)$ or $\gamma \sim 1/\exp(n)$.

We have shown that the QFC is asymptotically tight to the Nielsen's QCC, while the Bures distance (or the fidelity) is actually less stringent, because the Bures distance between two quantum states has a constant upper bound. To study topological phases,

we need to further incorporate the condition of geometric locality, which leads to the inequality (9) in Theorem 1 of the main text, where the lower bound of $\mathcal{C}_{\mathcal{N}}$ is asymptotically $\mathcal{C}_{\mathcal{N}} \geq \Omega(n)$. On the other hand, for the entanglement-based lower bound of $\mathcal{C}_{\mathcal{N}}$ in Theorem 2, we have

$$\mathcal{C}_{\mathcal{N}} \geq \Omega\left(\max_{k\in[1,n-1]} |S_k(\rho_1) - S_k(\rho_0)|\right). \tag{S27}$$

Therefore, for generating the (two-dimensional) Kitaev's toric code (which has area-law quantum entanglement) from a product state, this lower bound is $\mathcal{O}(L)$, where L is the system size. Since the known optimal QCC for the toric code is $\mathcal{O}(L)$ [S4], our entanglement-based bound is asymptotically tight for the toric code ground state, while the fidelity-based one overestimates the quantum circuit cost. On the other hand, for quantum states with volume-law quantum entanglement, we obtain similar lower bounds as in Theorem 1, that is, $\mathcal{O}(n)$. The (gapped) ground state of the bond-alternating XXZ spin chain in the main text has short-range quantum entanglement, whose quantum circuit depth / complexity is a constant, i.e., not scaling with n. The Von Neumann entropy (area-law scaling) of this ground state is also a constant. In this case, both the fidelity lower bound in (9) of Theorem 1 (after normalized by n in the kernel) and the entanglement lower bound in (S27) are asymptotically tight to the Nielsen's QCC. For more exotic and complicated quantum states, whose Nielsen's QCC may grow exponentially with n, both lower bounds [see Eq. (9) in Theorem 1 and Eq. (12) in Theorem 2] are no longer tight in this case. More elaborations will be made in future work to address such quantum states, as well as the information losses in the approximations of Nielsen's QCC.

IV. Complexity and scaling analysis of the method

As we have discussed in Sec. III, the quantum Fisher complexity is asymptotically tight to Nielsen's QCC, and contains information about topological properties, because the quantum Fisher information (QFI) reflects entanglement depth and critical behavior of the quantum state during the quantum path planning. In contrast, the Bures distance (and fidelity) is less stringent but more efficient in measurement and computation. For instance, in the numerical demonstrations of the main text, we use two-body reduced density matrices of constant size to cover the whole system, for which the computational or the measurement complexity in calculating the kernel is $\mathcal{O}(n)$. On the other hand, the entanglement-based kernel derived from Theorem 2 contains more topological information compared to the fidelity-based one. This is consistent with the knowledge that QFI and entanglement entropy are related to global properties of quantum states, which are *generically* hard to access through classical computation or measurement. For instance, one prominent approach based on classical measurement of quantum-state properties is the classical shadow tomography [S13], where nonlinear functional of the density matrix can be estimated from randomized Pauli measurement (classical shadows). In particular, QFI and Von Neumann entropy can be approximated [S14, S15], respectively, as a series expansion of polynomials of the density matrix, up to a prescribed precision. This involves estimations of high-order moments of the density matrix, which in general require exponentially many copies of the quantum states. Reference [S16] shows that polynomially many local measurements can be sufficient for estimating the second-order moment (or the Rényi entropy) if the quantum state posesses certain structures and properties, while it remains open for the Von Neumann entropy and the QFI, which deserves further investigations.

Therefore, we may conclude that topological order is hard to learn in the worst case. Alternatively, for those quantum states that are efficiently simulatable via tensor networks or matrix product states, the entanglement-entropy profile (which is related to the bond dimensions) is automatically stored and updated during the ground-state search process of the DMRG algorithm. This makes the entanglement-based kernel accessible efficiently. This point is also mentioned in the main text.

On the other hand, in case that our input data for the kernel are quantum states prepared on quantum devices, quantum algorithms may help to reduce the scaling and complexity of calculating the entanglement-based kernel. For instance, the quantum algorithm based on a Fourier series of the density matrix proposed in [S17] can estimate the Von Neumann entropy with $\mathcal{O}\left[\mathrm{poly}\left(\frac{1}{\epsilon},\, \frac{1}{\Lambda}\right)\right]$ copies of the quantum state and $\mathcal{O}\left[\mathrm{poly}\left(n, \frac{1}{\epsilon},\, \frac{1}{\Lambda}\right)\right]$ single- / two-qubit quantum gates, where ϵ is the approximation precision and Λ is the lower bound of non-zero eigenvalues of the density matrix. The algorithm provides a quantum speedup in case that Λ is a constant or is $\Omega\left[1/\mathrm{poly}\left(n\right)\right]$; Alternatively, the density-matrix-exponentiation (DME) algorithm [S18] can help to estimate the entanglement entropy of low-rank quantum states efficiently in the framework of Hamiltonian learning; etc. These results bring promise for quantum speedups in estimating the entanglement-based kernel within a quantum-classical hybrid learning framework. Moreover, estimating nonlinear functionals of the density matrix such as the QFI and the entanglement entropy efficiently on either classical, quantum, or hybrid platforms is still an active research area of quantum information and computation.

Finally, suppose that we have the number of samples (quantum states) N and a $N \times N$ kernel matrix, the (classical) computational complexity of the kernel PCA and the diffusion map (manifold learning) algorithm is $\mathcal{O}\left(N^3\right)$, which involves spectral analysis of the kernel matrix. This can be efficient for small datasets. For large datasets (e.g., $N > 100$), either the metric-MDS or the t-SNE for manifold learning is more suitable, the computational complexity of which is $\mathcal{O}(TN^2)$, where T is the number of iterations in the learning algorithm. (This completes the scaling analysis of our machine learning method.)

V. Effects of noise and random quantum circuits

Regarding potential effects of hardware noise, one may basically consider three scenarios.

The first scenario is that decoherence such as dephasing occurs during the quantum path planning or the quantum state preparation. For instance, the quantum Fisher information (i.e., the fidelity susceptibility) can scale as $\mathcal{O}\left(\mathrm{poly}(n)\, \mathrm{e}^{-\gamma n}\right)$ (under independent dephasing) or scale as $\mathcal{O}\left(\mathrm{poly}(n)\, \mathrm{e}^{-\gamma n^2}\right)$ (under correlated collective dephasing) for certain state evolution paths [S20], where γ is the noise strength. This may expain why the fidelity-based kernel is more fragile to noises. Generically, the presence of noise invalidates our original ansatz of unitary path planning, where the optimal distance is defined on a unitary-group manifold. Proper topological distance between noisy quantum states may require the analysis of the complexity of local quantum channels, a generalization of our current approach, which we leave for future work.

The second scenario is that, once the topologically ordered quantum state (e.g., Kitaev's toric code) is prepared, for instance, under quantum error mitigation or error correction, the established long-range entanglement will be robust to local decoherence. The Haar random unitaries uniformly apply random rotations or couplings to qubits, which is usually used for simulating and performance benchmarking of quantum information processing under strong hardware noise levels (e.g., depolarization). In this respect, the robustness against the Haar random two-qubit gates applied to the toric code in the main text (see Fig. 3 in the main text) indicates the superior performance of the entanglement-based kernel.

Finally, for an ensemble of random quantum states ρ (e.g., generated from random quantum circuits with the initial state ρ_0) described by a parametrized distribution p_θ, the ensemble-averaged kernel of the Nielsen's QCC is given by

$$\langle\, \mathcal{K}_{\mathcal{N}}\, \rangle_{p_\theta} = \int \mathrm{e}^{-\beta\, \mathcal{C}_{\mathcal{N}}(\rho_0,\, \rho)}\, p_\theta\left(\rho\right) \mathrm{d}\rho, \tag{S28}$$

which plays a role similar to the generating function [S21] of the (averaged) quantum circuit complexity,

$$\langle\, \mathcal{C}_{\mathcal{N}}\, \rangle_{p_\theta} = -\lim_{\beta \to 0} \frac{\partial}{\partial \beta} \langle\, \mathcal{K}_{\mathcal{N}}\, \rangle_{p_\theta}. \tag{S29}$$

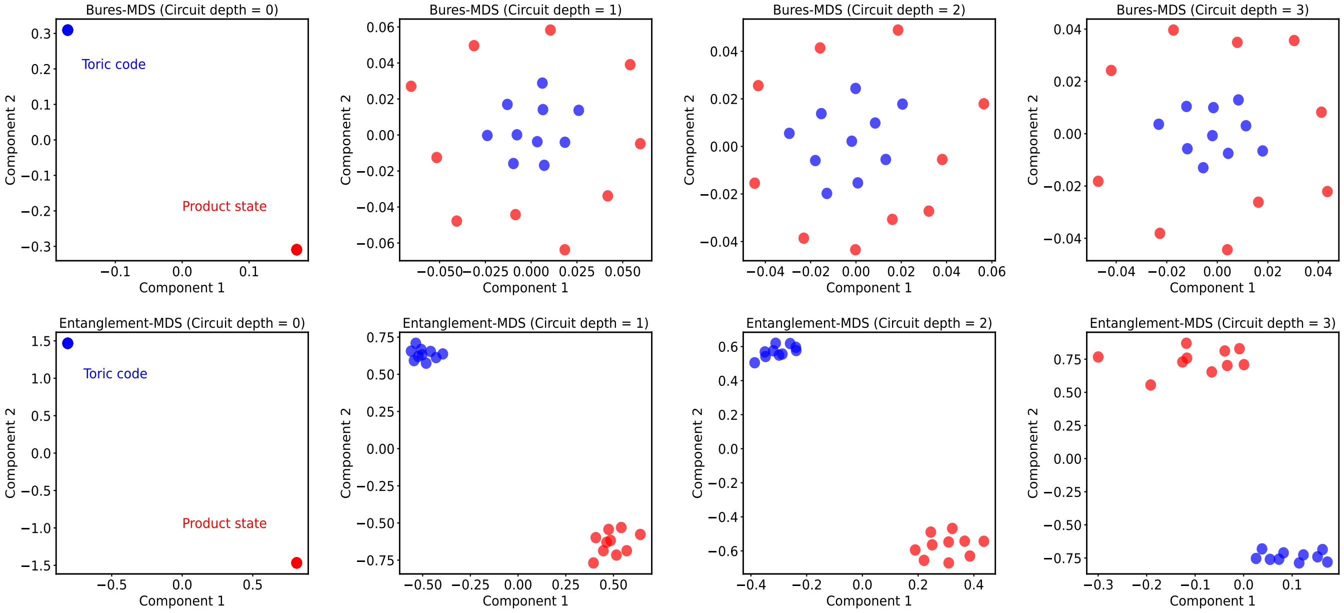

Figure S1. **Unsupervised manifold clustering of the ground state of Kitaev's toric code (blue dots in all sub-figures) and product states (red dots in all sub-figures) via the Bures distance (four sub-figures on the top) and entanglement distance (four sub-figures in the bottom) based metric-MDS, respectively.** Two-qubit Haar random gates [S19] of different circuit depth (Circuit depth $= 0, 1, 2, 3$) are applied repeatedly to both the toric code and the product state $|0\rangle^{\otimes n}$ (see the Methods seciton of the main text). We use $N = 20$ samples in total, with 10 samples of the toric code (blue dots) and 10 samples of the product state (red dots). We use $n = 32$ qubits (on a lattice of size 4×4 with toric boundary conditions; i.e., with a code distance of 4), and the DMRG (with the SVD cutoff 10^{-10} and the maximal energy error 10^{-10}) to solve for the ground state of the toric code and, at the same time, to extract the entanglement profile. We use n geometrically local 2-body reduced density matrices to cover the system and to calculate the local Bures distance in Eq. (9) of Theorem 1 in the main text. The result shows that, in the absence of noise (i.e. with circuit depth zero), both the Bures- and the entanglement-metrics perform well in clustering the toric code and the product states, while the entanglement-MDS is more robust to Haar random noises, indicating that the entanglement metric is a better choice in both the clustering performance and the interpretability.

This is similar to the action-propagator relation in generative modelling of Feynman paths via deep learning approach [S22], and can be useful in optimizing variational quantum circuits in various quantum machine learning tasks.

VI. Manifold learning of the toric code based on the metric-multidimensional scaling (metric-MDS)

Diffusion maps and kernel PCA that we have used in the main text involve a kernelization based on the distance metric, where the kernel is used to construct a conditional probability in the diffusion map (as well as in the t-SNE algorithm) and is directly diagonalized in the kernel PCA. While this kernelization may complicate the understanding of the distance metrics, we can use an alternative manifold learning called the metric-multidimensional scaling (metric-MDS) [S23, S24], where high-dimensional manifold of the dataset with the Bures or entanglement distance metric can be embedded into a low dimensional Euclidean space, while preserving the original distance relations of the data manifold *without involving a kernel*. This can be realized, for instance, by minimizing a stress function (cost function) iteratively [S23, S24]. *The metric-MDS does not require a kernel, but utilizes the distance metric of the manifold directly as in the Isomap [S25].* In Fig. S1, we plot the comparison of the Bures-MDS and

the entanglement-MDS manifold learning for the toric code. We find that, in the absence of noise (i.e. with circuit depth zero), both the Bures- and the entanglement-metrics perform well in clustering the toric code and the product states. After applying two-qubit Haar random local quantum gates, the entanglement-MDS is more robust in the clustering performance, indicating that the entanglement metric is a better choice accounting for both the clustering performance and the interpretability (i.e., it contains more topological information). More details can be found in the caption of Fig. S1.

[S1] M. M. Taddei, B. M. Escher, L. Davidovich, and R. L. de Matos Filho, "Quantum Speed Limit for Physical Processes," Phys. Rev. Lett. **110**, 050402 (2013).

[S2] M. A. Nielsen and I. L. Chuang, *Quantum Computation and Quantum Information* (Cambridge University Press, Cambridge, England, 2012).

[S3] J. Eisert, "Entangling Power and Quantum Circuit Complexity," Phys. Rev. Lett. **127**, 020501 (2021).

[S4] S. Bravyi, M. B. Hastings, and F. Verstraete, "Lieb-Robinson Bounds and the Generation of Correlations and Topological Quantum Order," Phys. Rev. Lett. **97**, 050401 (2006).

[S5] S. Bravyi, "Upper bounds on entangling rates of bipartite hamiltonians," Phys. Rev. A **76**, 052319 (2007).

[S6] A. Hutter and S. Wehner, "Almost All Quantum States Have Low Entropy Rates for Any Coupling to the Environment," Phys. Rev. Lett. **108**, 070501 (2012).

[S7] K. Van Acoleyen, M. Mariën, and F. Verstraete, "Entanglement Rates and Area Laws," Phys. Rev. Lett. **111**, 170501 (2013).

[S8] M. Mariën, K. M. R. Audenaert, K. Van Acoleyen, and F. Verstraete, "Entanglement rates and the stability of the area law for the entanglement entropy," Communications in Mathematical Physics **346**, 35–73 (2016).

[S9] S. Bachmann, S. Michalakis, B. Nachtergaele, and R. Sims, "Automorphic Equivalence within Gapped Phases of Quantum Lattice Systems," Commun. Math. Phys. **309**, 835–871 (2012).

[S10] T. Albash and D. A. Lidar, "Adiabatic quantum computation," Rev. Mod. Phys. **90**, 015002 (2018).

[S11] W.-L. You, Y.-W. Li, and S.-J. Gu, "Fidelity, dynamic structure factor, and susceptibility in critical phenomena," Phys. Rev. E **76**, 022101 (2007).

[S12] K. Gietka, F. Metz, T. Keller, and J. Li, "Adiabatic critical quantum metrology cannot reach the Heisenberg limit even when shortcuts to adiabaticity are applied," Quantum **5**, 489 (2021).

[S13] H.-Y. Huang, R. Kueng, and J. Preskill, "Predicting many properties of a quantum system from very few measurements," Nat. Phys. **16**, 1050–1057 (2020).

[S14] A. Rath, C. Branciard, A. Minguzzi, and B. Vermersch, "Quantum Fisher Information from Randomized Measurements," Phys. Rev. Lett. **127**, 260501 (2021).

[S15] B. Vermersch, A. Rath, B. Sundar, C. Branciard, J. Preskill, and A. Elben, "Enhanced Estimation of Quantum Properties with Common Randomized Measurements," PRX Quantum **5**, 010352 (2024).

[S16] B. Vermersch, M. Ljubotina, J. I. Cirac, P. Zoller, M. Serbyn, and L. Piroli, "Many-Body Entropies and Entanglement from Polynomially Many Local Measurements," Phys. Rev. X **14**, 031035 (2024).

[S17] Y. Wang, B. Zhao, and X. Wang, "Quantum algorithms for estimating quantum entropies," Phys. Rev. Appl. **19**, 044041 (2023).

[S18] S. Lloyd, M. Mohseni, and P. Rebentrost, "Quantum principal component analysis," Nat. Phys. **10**, 631 (2014).

[S19] J. Hauschild and F. Pollmann, "Efficient numerical simulations with Tensor Networks: Tensor Network Python (TeNPy)," SciPost Phys. Lect. Notes , 5 (2018), code available from `https://github.com/tenpy/tenpy`, arXiv:1805.00055.

[S20] Y. Che, J. Liu, X.-M. Lu, and X. Wang, "Multiqubit matter-wave interferometry under decoherence and the Heisenberg scaling recovery," Phys. Rev. A **99**, 033807 (2019).

[S21] M. Miyaji, S.-M. Ruan, S. Shibuya, and K. Yano, "Universal Time Evolution of Holographic and Quantum Complexity," arxiv:2507.23667 (2025).

[S22] Y. Che, C. Gneiting, and F. Nori, "Estimating the Euclidean quantum propagator with deep generative modeling of Feynman paths," Phys. Rev. B **105**, 214205 (2022).

[S23] E. Demaine, A. Hesterberg, F. Koehler, J. Lynch, and J. Urschel, "Multidimensional Scaling: Approximation and Complexity," in *Proceedings of the 38th International Conference on Machine Learning*, Proceedings of Machine Learning Research, Vol. 139, edited by Marina Meila and Tong Zhang (PMLR, 2021) pp. 2568–2578.

[S24] F. Pedregosa, G. Varoquaux, A. Gramfort, V. Michel, B. Thirion, O. Grisel, M. Blondel, P. Prettenhofer, R. Weiss, V. Dubourg, J. Vanderplas, A. Passos, D. Cournapeau, M. Brucher, M. Perrot, and E. Duchesnay, "Scikit-learn: Machine learning in Python," Journal of Machine Learning Research **12**, 2825–2830 (2011).

[S25] J. B. Tenenbaum, V. De Silva, and J. C. Langford, "A global geometric framework for nonlinear dimensionality reduction," Science **290**, 2319–2323 (2000).
