# Supplementary material for: Quantum circuit complexity and unsupervised machine learning of topological order
Source: Nat Commun. 2026 Apr 8;17:5179. doi: 10.1038/s41467-026-71283-5 (PMC13254324; doi:10.1038/s41467-026-71283-5)
Supplement: Supplementary file 2 — Transparent Peer Review file [file 41467_2026_71283_MOESM2_ESM.pdf]

nature portfolio

Peer Review File

Quantum circuit complexity and unsupervised machine learning of topological order

Corresponding Author: Dr Yanming Che

This file contains all reviewer reports in order by version, followed by all author rebuttals in order by version.

Version 0:

Reviewer comments:

Reviewer #1

(Remarks to the Author)
In this manuscript, the author discussed the utility of quantum circuit complexity as a key concept in distinguishing topological phase and trivial phase of many-body ground states. The main contribution includes two lower bounds of quantum circuit complexity, which can be experimentally measured in practical quantum systems. Associated with these lower bounds, the authors proposed two kernel functions which can be used to classify topological phase and trivial phase in an unsupervised manner.

I think the idea of connecting Nilsen's quantum circuit complexity with many-body phase classification is quite interesting. However, I have concerns regarding the novelty of the methods used to classify different phases. As part of the main results in this paper, the kernel methods used to classify topological phase and trivial phases are very similar to the approach developed in H-Y Huang et al. "Provably efficient machine learning for quantum many-body problems". Especially, the fidelity-based kernel is quite close to shadow kernel. Hence, the numerical results of successful classification are not surprised.

Two other comments are: 1. I am not sure how tight those two lower bounds of quantum circuit complexity are, especially for those many-body ground states discussed in the paper; 2. if I understand correctly, the entanglement-based kernel requires the estimation of subsystem entanglement entropy of all possible sizes, which is challenging to measure in scalable systems especially when it cannot be classically simulated by MPS.

Based on the above comments, I believe this manuscript fails to satisfy the high criteria of Nature Communications.

(Remarks on code availability)

Reviewer #2

(Remarks to the Author)
In the present paper the Authors focus on the task of identifying topological orders of many-body states with quantum algorithmic approach, namely building corresponding cirquit and evaluating its complexity. The paper proposes a new theoretical framework for unsupervised machine learning of topological quantum order by leveraging quantum circuit complexity (QCC), by providing a proof of several key theorems. The authors reinterpret the notion of topological equivalence between quantum states in terms of constant-depth quantum circuits. This connects machine learning kernels with a fundamental measure of quantum complexity for the two kernel constructions - fidelity-based kernel and entanglement-based kernel. Theorems establish lower bounds on QCC via Bures distance (fidelity changes) and entanglement changes. The Authors demonstrate that the proposed kernels can be efficiently computed in simulations like DMRG and potentially measured experimentally using classical shadow tomography. Examples contain bond-alternating XXZ spin chain and toric code. The paper is sound and represents an important step in building interpretable unsupervised machine learning techniques for identification of topological orders. The analytical proofs of theorems are clearly written. The presented figures explain well the main findings of numerical simulations and provide a clear comparison of two proposed kernels. I would recommend paper for publication after the following questions and comments are clarified:
1. How well are presented kernels expected to scale for larger systems? In other words, whether the proposed kernel-based

methods will be practically applicable for large condensed matter systems of interest?
2. The entanglement-based kernel is shown to be more robust against two-qubit random noise. How do both kernels behave under realistic hardware noise models (e.g., correlated errors, decoherence)?
3. In Fig.2, panels (c) and (d) the axes labels are indicated only in the caption. Could they be indicated also on the axes? In addition, both plots contain a single point of the yellow-light-green color, that stays in the middle between phases. This point corresponds approximately to J2/J1=1.5 . If more dense grid is taken for J2/J1 ratios, would it result in symmetry-broken phase being smoothly connected to one of the topo/trivial phases in the axes coordinates? This question requires clarification as one might expect the same problems of intersecting areas as one finds in principal component analysis classification of phases.

(Remarks on code availability)
The run of code provides the same output as shown in figures. However, the code does not contain the readme file that would clearly indicate a structure and purpose of particular files / classes and functions. At the same time, file names and short readme files with instructions to run explain the general idea behind the code. In addition, not all the functions and classes implemented in the code have their clear descriptions in the form of docstrings or in readme file. My overall evaluation is that the code is good for reproducibility purposes for data shown in paper, but has limitations in re-use as it requires extra time to understand its internal structure.

Reviewer #3

(Remarks to the Author)
In this reviewed manuscript "Quantum circuit complexity and unsupervised machine learning of topological order", the authors explore how to build interpretable and efficient unsupervised learning model for identifying topological order, with a focus on the quantum circuit complexity. The authors prove two theorems that link Nielsen's geometric definition of quantum circuit complexity to fidelity variation and entanglement entropy differences between quantum states. From these relations, they derive two practical kernels (fidelity-based and entanglement-based kernels) to capture the topological similarity between states. They also carry out numerical experiments on classifying phases of XXZ spin chain, ground states of Kitaev's toric code to support their theoretical findings. A connection to shadow kernel learning is discussed.

Unsupervised learning topological phases of matter is a challenging task. This work makes a conceptual step by tying informational distances to quantum phase classification, providing an interpretable bridge between complexity theory and practical machine learning. I think this is an interesting paper and a timely contribution to both quantum information and condensed matter communities. Given that the manuscript is original, conceptually well-motivated, and technically sounds, I thus recommend the acceptance of this work in Nature Communications after the following comments/suggestions are carefully addressed:

1)The toric code test with random unitaries suggests robustness of the entanglement kernel. It would be helpful to quantify how classification performance degrades with increasing noise levels. This would make the claims of robustness more concrete.

2)The numerical demonstrations involve systems of 101 spins (XXZ chain) and 32 qubits (toric code). While this is reasonable for proof-of-principle, it would be helpful to comment more on the scalability.

3)The authors may consider citing the following works, which report related advances and would enrich the discussion.
1. npj Quantum Information 8, 116(2022);
2.Fundamental Research 4, 1086(2024).

4)Some typos:
Line 72 : "a a suitable distance" --> "a suitable distance".
Line 158: "permmutations" --> "permutations".

(Remarks on code availability)

Version 1:

Reviewer comments:

Reviewer #1

(Remarks to the Author)
Thanks to the authors for their response to my comments. I appreciate that the main contribution of this work is to offer a new perspective and framework for the unsupervised learning of topological quantum phases, which enhances the interpretability of such classifications. Nevertheless, I remain unconvinced that these contributions justify publication in Nature Communications. My primary concern is that the link drawn between quantum circuit complexity and topological phases does not, in my view, yield fundamentally new insight. As noted in the authors' response, fidelity-based methods here share

similarities with earlier shadow-based approaches. Likewise, entanglement-based methods are not wholly novel, given the established understanding that subsystem entanglement entropy encodes topological features of quantum states.
In summary, while this work introduces a valuable perspective on unsupervised classification of topological phases, it lacks demonstrative results showing that the proposed approach surpasses prior methods in computational efficiency or experimental feasibility.

(Remarks on code availability)

Reviewer #2

(Remarks to the Author)
In the revised manuscript, the Authors addressed all question and requests provided by the referees and significantly improved a manuscript in the number of subtle points. In particular, the discussion about the scaling of approach with sizes and the performance with the noise was significantly improved. Based on this evaluation and the timely appearance of these results, as was pointed out by the third Referee, I recommend the paper for publication in Nature Communications journal.

(Remarks on code availability)
The Authors improved the clarity and reusability of the code by providing explanation files within each section, as was requested.

Reviewer #3

(Remarks to the Author)
The authors have addressed all of my concerns in the revised manuscript. I thus recommend the publication of this work in Nature Communications.

(Remarks on code availability)

Open Access This Peer Review File is licensed under a Creative Commons Attribution 4.0 International License, which permits use, sharing, adaptation, distribution and reproduction in any medium or format, as long as you give appropriate credit to the original author(s) and the source, provide a link to the Creative Commons license, and indicate if changes were made.
In cases where reviewers are anonymous, credit should be given to 'Anonymous Referee' and the source.
The images or other third party material in this Peer Review File are included in the article’s Creative Commons license, unless indicated otherwise in a credit line to the material. If material is not included in the article’s Creative Commons license and your intended use is not permitted by statutory regulation or exceeds the permitted use, you will need to obtain permission directly from the copyright holder.
To view a copy of this license, visit https://creativecommons.org/licenses/by/4.0/

In the following we provide a point-by-point response to the reviewers' comments and suggestions:

==

Response to Reviewer #1:

Referee's comment (general): In this manuscript, the author discussed the utility of quantum circuit complexity as a key concept in distinguishing topological phase and trivial phase of many-body ground states. The main contribution includes two lower bounds of quantum circuit complexity, which can be experimentally measured in practical quantum systems. Associated with these lower bounds, the authors proposed two kernel functions which can be used to classify topological phase and trivial phase in an unsupervised manner.

I think the idea of connecting Nilsen's quantum circuit complexity with many-body phase classification is quite interesting. However, I have concerns regarding the novelty of the methods used to classify different phases. As part of the main results in this paper, the kernel methods used to classify topological phase and trivial phases are very similar to the approach developed in H-Y Huang et al. "Provably efficient machine learning for quantum many-body problems". Especially, the fidelity-based kernel is quite close to shadow kernel. Hence, the numerical results of successful classification are not surprised.

Reply:

We thank the reviewer for the positive comments on our main contribution in the manuscript, in particular, for acknowledging that "the idea of connecting Nielsen's quantum circuit complexity with many-body phase classification is quite interesting".

We agree with the reviewer that our usage of the kernel PCA to classify toric code and trivial phases, in particular, the fidelity-based kernel, shares some similarities with the approach developed in H-Y Huang et al. [1].

However, let us clarify that, *as Reviewer #2 and Reviewer #3 have also pointed out,* the focus of our manuscript is to make a conceptual step toward solving the challenging task of interpretable machine learning of many-body topological quantum order. The novelty is to link quantum algorithms and informational distances (Nielsen's quantum circuit complexity) with topological quantum phase classification, building a bridge between complexity theory and interpretable machine learning, where interpretability and generalizability are of central concern.

In our work, such an intrinsic distance metric (Nielsen's quantum circuit complexity and its substitutes) is a core component for (unsupervised) manifold learning of topological order, where the data is assumed to be distributed on a manifold characterized by this distance metric. Once this intrinsic distance of data manifold is established, the following specific algorithm of manifold learning can be diverse, including kernel or non-kernel methods. For instance, in the diffusion map (used in generating Fig. 2 of our main text) and the t-SNE algorithms, a kernel built from a distance metric is used to construct a probabilistic graph on the data manifold; In the kernel PCA (for generating Fig. 3 and is also adopted in [1]), data points are embedded into the space of leading eigen-components of the kernel matrix. Regarding non-kernel methods, such as the Isomap and the metric-multidimensional scaling (metric-MDS), a distance matrix of the data manifold is already sufficient for the unsupervised clustering (Now we added a metric-MDS classification of the toric code and product state in Sec. VI of the Supplementary Information, as an alternatively non-kernel approach compared to the kernel PCA in Fig. 3).

The classical shadow kernel in [1] is formulated in a very elegant manner but seems to lack explicit interpretability, in particular, when applied to clustering topological order (for instance, from the primitive notion of topological equivalence). We agree with the reviewer that our fidelity-based kernel can be not new in the sense that it is similar to the shadow kernel [1] (which we already pointed out in the manuscript), while it is derived from a manifold distance metric for topological order, i.e., the Nielsen's QCC. This is an important new insight beyond the paper [1], and can be helpful in increasing the interpretability of the classical shadow kernel. Moreover, an

alternative distance / kernel approximated from the Nielsen’s QCC is entanglement-based, which arguably is the more generalizable and more interpretable one for clustering topological phases. This has not yet been proposed in [1] or elsewhere.

Other improved distance approximations (compared to the fidelity-based one) could be discovered in the future. One possible choice can be the quantum Fisher complexity (QFC), because quantum Fisher information also reflects the entanglement depth of quantum states, and we now show in the added Sec. III of the Supplementary Information that the QFC has the same scaling behaviour as Nielsen's QCC.

Moreover, our method is, in principle, not restricted to only clustering topological and trivial phases. There can be multiple topologically distinct phases on the data manifold to be addressed. The method serves to distinguish any two quantum many-body states that have different quantum circuit complexities or long-range entanglement structures.

In the revised manuscript, we now added more elaborations to clarify these points and the focus of our work (see the blue marked sentences in the Abstract, Introduction, Methods, etc.). Following the reviewer’s suggestion, we also added tightness analyses of the bounds in Theorems 1 and 2; as well as the scaling analysis of the method (see Sec. III and Sec. IV, respectively, in the Supplementary Information, which are also mentioned / referenced in the main text.). A non-kernel manifold learning of the toric code is performed in Sec. VI of the Supplementary Information, which gives similar results as the kernel PCA in Fig. 3.

We hope that these revisions can address your comments and can remove your concern satisfactorily.

Thank you very much for your constructive comments and suggestions which have been very helpful in improving the manuscript significantly.

<u>Comment 1:</u> I am not sure how tight those two lower bounds of quantum circuit complexity are, especially for those many-body ground states discussed in the paper;

<u>Reply:</u> Many thanks for this constructive comment. Now in the revised version, we have added "Sec. III: Asymptotic and tightness analysis of the bounds in Theorems 1 and 2" in the Supplementary Information to address this comment.

<u>Comment 2:</u> If I understand correctly, the entanglement-based kernel requires the estimation of subsystem entanglement entropy of all possible sizes, which is challenging to measure in scalable systems especially when it cannot be classically simulated by MPS.

<u>Reply:</u> We understand the reviewer's concern. Indeed, the entanglement entropy profile requires the estimation of subsystem entanglement entropy of all possible sizes (at least half system size due to the symmetry). This is a challenging task in the worst case, which indicates the hardness of learning topological order in the worst case. Efficient tensor-network states and MPS, or quantum algorithms for certain quantum states can make the measurement of entanglement entropy more accessible. This is still an active research area of quantum information and computation.

Now we added elaborative discussions about this comment in "Sec. IV: Complexity and scaling analysis of the method" of the Supplementary Information.

=========

References:

[1]. H.-Y. Huang, R. Kueng, G. Torlai, V. V. Albert, and J. Preskill, "Provably efficient machine learning for quantum many-body problems," Science 377, eabk3333 (2022).

===

Response to Reviewer #2:

Referee's comment (general):

In the present paper the Authors focus on the task of identifying topological orders of many-body states with quantum algorithmic approach, namely building corresponding circuit and evaluating its complexity. The paper proposes a new theoretical framework for unsupervised machine learning of topological quantum order by leveraging quantum circuit complexity (QCC), by providing a proof of several key theorems. The authors reinterpret the notion of topological equivalence between quantum states in terms of constant-depth quantum circuits. This connects machine learning kernels with a fundamental measure of quantum complexity for the two kernel constructions - fidelity-based kernel and entanglement-based kernel. Theorems establish lower bounds on QCC via Bures distance (fidelity changes) and entanglement changes. The Authors demonstrate that the proposed kernels can be efficiently computed in simulations like DMRG and potentially measured experimentally using classical shadow tomography. Examples contain bond-alternating XXZ spin chain and toric code. The paper is sound and represents an important step in building interpretable unsupervised machine learning techniques for identification of topological orders. The analytical proofs of theorems are clearly written. The presented figures explain well the main findings of numerical simulations and provide a clear comparison of two proposed kernels. I would recommend paper for publication after the following questions and comments are clarified:

Reply:

We thank the reviewer for the positive comments, highlighting that "The paper proposes a new theoretical framework for unsupervised machine learning of topological quantum order by leveraging quantum circuit complexity (QCC), by providing a proof of several key theorems", and "represents an important step in building interpretable unsupervised machine learning techniques for identification of topological orders".
We thank the reviewer for the prospect of recommendation of our manuscript for publication.

Below we provide a point-to-point response to the very constructive comments from the reviewer, which have been very helpful in improving the manuscript significantly.

Comment 1: How well are presented kernels expected to scale for larger systems? In other words, whether the proposed kernel-based methods will be practically applicable for large condensed matter systems of interest?

Reply: We thank the reviewer very much for pointing this scaling aspect out, which was missing in our original manuscript. Now we added an elaborative discussion on the scaling analysis (with respect to the system size as well as the sample size) of estimating the kernels and of the method. Please see "Sec. IV: Complexity and scaling analysis of the method" in the Supplementary Information (which is also referenced / mentioned in the main text).

Comment 2: The entanglement-based kernel is shown to be more robust against two-qubit random noise. How do both kernels behave under realistic hardware noise models (e.g., correlated errors, decoherence)?

Reply: We thank the reviewer for the constructive comment. Noises can happen in the quantum state preparation or can be simulated by applying random quantum circuits. Now we added discussions on the noise effects considering potentially three scenarios. Please see "Sec. V: Effects of noise and random quantum circuits" in the Supplementary Information (which is also referenced / mentioned in the main text).

Comment 3: In Fig.2, panels (c) and (d) the axes labels are indicated only in the caption. Could they be indicated also on the axes?
In addition, both plots contain a single point of the yellow-light-green color, that stays in the middle between phases. This point corresponds approximately to J2/J1=1.5 . If more dense grid is taken for J2/J1 ratios, would it result in symmetry-broken phase being smoothly connected to one of the topo/trivial phases in the axes coordinates? This question requires

clarification as one might expect the same problems of intersecting areas as one finds in principal component analysis classification of phases.

<u>Reply:</u> We thank the reviewer for pointing it out. Now we added the axes labels in panels (c) and (d). The outlier point corresponding to J2/J1=1.5 is the (gapless) critical phase between the symmetry-broken and topological phases. This is due to the finite-size effect and the resulted finite-width of the critical region. Now we replotted Figure 2 with a larger system size (number of qubits n = 151), where the clustering performance is improved and the size of each phase-cluster becomes smaller compared to the orginal plots with n = 101.

<u>Remarks on code availability:</u>

The run of code provides the same output as shown in figures. However, the code does not contain the readme file that would clearly indicate a structure and purpose of particular files / classes and functions. At the same time, file names and short readme files with instructions to run explain the general idea behind the code. In addition, not all the functions and classes implemented in the code have their clear descriptions in the form of docstrings or in readme file. My overall evaluation is that the code is good for reproducibility purposes for data shown in paper, but has limitations in re-use as it requires extra time to understand its internal structure.

<u>Reply:</u> We thank the reviewer very much for kindly pointing this out. In a later submission to the Code Ocean, we included a Readme text for all the code files, describing the run steps to reproduce the figures in our manuscript, which will be helpful in improving the readability and the re-usability of our code. Now we also add a Readme text for each code file in this resubmission.

==

Response to Reviewer #3:

Referee's comment (general):

In this reviewed manuscript "Quantum circuit complexity and unsupervised machine learning of topological order", the authors explore how to build interpretable and efficient unsupervised learning model for identifying topological order, with a focus on the quantum circuit complexity. The authors prove two theorems that link Nielsen's geometric definition of quantum circuit complexity to fidelity variation and entanglement entropy differences between quantum states. From these relations, they derive two practical kernels (fidelity-based and entanglement-based kernels) to capture the topological similarity between states. They also carry out numerical experiments on classifying phases of XXZ spin chain, ground states of Kitaev's toric code to support their theoretical findings. A connection to shadow kernel learning is discussed.

Unsupervised learning topological phases of matter is a challenging task. This work makes a conceptual step by tying informational distances to quantum phase classification, providing an interpretable bridge between complexity theory and practical machine learning. I think this is an interesting paper and a timely contribution to both quantum information and condensed matter communities. Given that the manuscript is original, conceptually well-motivated, and technically sounds, I thus recommend the acceptance of this work in Nature Communications after the following comments/suggestions are carefully addressed:

Reply:

We thank the reviewer for the positive comments, in particular, for highlighting our focus on quantum circuit complexity and on "providing an interpretable bridge between complexity theory and practical machine learning", and for the (conditional) recommendation of acceptance of our work.

Below we provide a point-to-point response to the very constructive comments from the reviewer, which have been very helpful in improving the manuscript significantly.

<u>Comment 1:</u> The toric code test with random unitaries suggests robustness of the entanglement kernel. It would be helpful to quantify how classification performance degrades with increasing noise levels. This would make the claims of robustness more concrete.

<u>Reply:</u> We thank the reviewer very much for kindly pointing this out, which was missing in the original manuscript. Now we added sub-figures which plot the silhouette coefficient / score, a measure of the clustering performance. These plots show clearly that the performance degradation of the entanglement-based approach is much slower as the noise level increases. Please see Fig. 3 (b) and (d).

<u>Comment 2:</u> The numerical demonstrations involve systems of 101 spins (XXZ chain) and 32 qubits (toric code). While this is reasonable for proof-of-principle, it would be helpful to comment more on the scalability.

<u>Reply:</u> We understand the reviewer's concern. Now we added more comments and discussions on the scaling analysis of the method in "Sec. IV: Complexity and scaling analysis of the method" of the Supplementary Information (which is also referenced / mentioned in the main text). Many thanks for pointing this out.

<u>Comment 3:</u> The authors may consider citing the following works, which report related advances and would enrich the discussion.
1. npj Quantum Information 8, 116(2022);
2.Fundamental Research 4, 1086(2024).

<u>Reply:</u> Thank you for pointing out these relevant and important works. Now we cite these works in the updated manuscript.

<u>Comment 4:</u> Some typos:
Line 72 : "a a suitable distance" --> "a suitable distance".
Line 158: "permmutations" --> "permutations".

<u>Reply:</u> It is greatly appreciated. Now these typos have been corrected. Please see the manuscript.

==

In summary, we believe that we have addressed all the reviewers' concerns and comments. We hope that now the paper is suitable for publication in Nature Communications.

Response to reviewers' comments and recommendations in the second round of review:

===

Response to Reviewer #1:

<u>Referee's comments:</u> Thanks to the authors for their response to my comments. I appreciate that the main contribution of this work is to offer a new perspective and framework for the unsupervised learning of topological quantum phases, which enhances the interpretability of such classifications. Nevertheless, I remain unconvinced that these contributions justify publication in Nature Communications. My primary concern is that the link drawn between quantum circuit complexity and topological phases does not, in my view, yield fundamentally new insight. As noted in the authors' response, fidelity-based methods here share similarities with earlier shadow-based approaches. Likewise, entanglement-based methods are not wholly novel, given the established understanding that subsystem entanglement entropy encodes topological features of quantum states. In summary, while this work introduces a valuable perspective on unsupervised classification of topological phases, it lacks demonstrative results showing that the proposed approach surpasses prior methods in computational efficiency or experimental feasibility.

<u>Reply:</u>

We thank the referee for appreciating our main contribution in this paper that "offer a new perspective and framework for the unsupervised learning of topological quantum phases, which enhances the interpretability of such classifications"; and that

“introduces a valuable perspective on unsupervised classification of topological phases”.

The new insight we provide here is a quantum algorithmic information paradigm to understand and, more importantly, to generalize unsupervised (classical and quantum) machine learning of quantum many-body states. It is not restricted to interpretable phase clustering, but also can be applied to generative modelling of (topological) quantum states via optimized / minimal quantum circuits (i.e., quantum program) from a reference quantum state.

The Nielsen’s quantum circuit complexity itself has been widely studied in quantum information and quantum field theories; Separately, the same happened to quantum Fisher information and Bures distance (see the Introduction section of our paper). However, the bridge to these concepts in a provable manner (Theorem 1 in our paper), and thus, the connection to the shadow kernel learning in the literature, has not been established elsewhere.

Additionally, the geometric locality of the reduced density matrix is a new ingredient in our fidelity-based model, while the shadow kernel does not require it explicitly. We find that the geometric locality will boost the clustering performance, in particular, when the system size becomes large (for instance, when the number of qubits is n = 151 in the bond-alternating XXZ spin model in our paper).

Regarding the sample efficiency and the computational efficiency, we have provided an elaborated analysis in Sec. IV of the

Supplementary Information (SI). (1) For the scaling of the method with respect to the number of qubits n, the entanglement-based approach is efficient for quantum states that are simulated by MPS or tensor networks, and is experimentally feasible under efficient quantum measurement and quantum estimation protocols (e.g., the density matrix exponentiation or quantum algorithms for entanglement entropy estimations; see our discussions in the SI). (2) For the scaling of the method with respect to the number of samples *N*, the kernel-based methods (including the shadow kernel learning) are O(*N*^3), while manifold learning directly based on the informational distances (without a kernelization) such as the metric-MDS (see Fig. S1 in the SI) is more efficient, scaling with O(*N*^2). This is superior for large data set. We have provided such discussions in the SI (see Sev. IV) and the Methods section.

These are provable results that all demonstrative experiments should obey.

In summary, we thank the referee for the appreciation and the positive comments on our main contribution in this work, and appreciate the reviewer’s significant contributions in the review process. We clarify new insights and efficiency of our method in response to other comments of the reviewer. In this paradigm of quantum algorithmic information, we hope our work would pave the way toward an interpretable, generalizable, and efficient (both classically and quantumly) unsupervised machine learning for topological order.

==

Response to Reviewer #2:

<u>Referee's comments:</u> In the revised manuscript, the Authors addressed all question and requests provided by the referees and significantly improved a manuscript in the number of subtle points. In particular, the discussion about the scaling of approach with sizes and the performance with the noise was significantly improved. Based on this evaluation and the timely appearance of these results, as was pointed out by the third Referee, I recommend the paper for publication in Nature Communications journal.

(Remarks on code availability): The Authors improved the clarity and reusability of the code by providing explanation files within each section, as was requested.

<u>Reply:</u>

We thank the reviewer for the positive comments and the recommendation of our paper for publication. We appreciate the reviewer's significant contributions in the review process.

==

Response to Reviewer #3:

<u>Referee's comments:</u> The authors have addressed all of my concerns in the revised manuscript. I thus recommend the publication of this work in Nature Communications.

Reply:

We thank the reviewer for the positive comments and the recommendation of our paper for publication. We appreciate the reviewer's significant contributions in the review process.
